# Protocol: Implementation and evaluation of an adolescent-mediated intervention to improve glycemic control and diabetes self-management among Samoan adults

**DOI:** 10.1371/journal.pone.0279084

**Published:** 2023-02-16

**Authors:** Nicola L. Hawley, Anna C. Rivara, Joshua Naseri, Kitiona Faumuina, Noelle Potoa’e-Solaita, Francine Iopu, Mata’uitafa Faiai, Eminoni Naveno, Susie Tasele, Temukisa Lefale, Ryan Lantini, Jenna C. Carlson, Tracy L. Rabin, Penny Semaia, Phyllis Mugadza, Rochelle K. Rosen

**Affiliations:** 1 Department of Chronic Disease Epidemiology, Yale School of Public Health, New Haven, CT, United States of America; 2 Obesity, Lifestyle and Genetic Adaptations Study Group, Pago Pago, American Samoa; 3 Department of Public Health, Portland State University, Portland, OR, United States of America; 4 Department of Health, Pago Pago, American Samoa; 5 Center for Behavioral and Preventative Medicine, The Miriam Hospital, Providence, RI, United States of America; 6 Department of Biostatistics, School of Public Health, University of Pittsburgh, Pittsburgh, PA, United States of America; 7 Department of Human Genetics, School of Public Health, University of Pittsburgh, Pittsburgh, PA, United States of America; 8 Department of Internal Medicine, Yale University School of Medicine, New Haven, CT, United States of America; 9 Athletic Department, University of Pittsburgh, Pittsburgh, PA, United States of America; PhD, PLOS, UNITED KINGDOM

## Abstract

**Background:**

Diagnoses of Type 2 Diabetes in the United States have more than doubled in the last two decades. One minority group at disproportionate risk are Pacific Islanders who face numerous barriers to prevention and self-care. To address the need for prevention and treatment in this group, and building on the family-centered culture, we will pilot test an adolescent-mediated intervention designed to improve the glycemic control and self-care practices of a paired adult family member with diagnosed diabetes.

**Methods:**

We will conduct a randomized controlled trial in American Samoa among n = 160 dyads (adolescent without diabetes, adult with diabetes). Adolescents will receive either a six-month diabetes intervention or a leadership and life skills-focused control curriculum. Aside from research assessments we will have no contact with the adults in the dyad who will proceed with their usual care. To test our hypothesis that adolescents will be effective conduits of diabetes knowledge and will support their paired adult in the adoption of self-care strategies, our primary efficacy outcomes will be adult glycemic control and cardiovascular risk factors (BMI, blood pressure, waist circumference). Secondarily, since we believe exposure to the intervention may encourage positive behavior change in the adolescent themselves, we will measure the same outcomes in adolescents. Outcomes will be measured at baseline, after active intervention (six months post-randomization) and at 12-months post-randomization to examine maintenance effects. To determine potential for sustainability and scale up, we will examine intervention acceptability, feasibility, fidelity, reach, and cost.

**Discussion:**

This study will explore Samoan adolescents’ ability to act as agents of familial health behavior change. Intervention success would produce a scalable program with potential for replication in other family-centered ethnic minority groups across the US who are the ideal beneficiaries of innovations to reduce chronic disease risk and eliminate health disparities.

## Introduction

The number of individuals diagnosed with Type 2 Diabetes in the United States (US) has more than doubled since 2000 to over 30 million, with an additional 84.1 million living with prediabetes [1]. This burden falls disparately on racial and ethnic minority groups, who have a higher prevalence of diabetes, a greater burden of disease, and also experience a higher rate of complications [2]. One minority group at particular risk is Pacific Islanders (PIs). Estimates of diabetes prevalence among PIs in the US range from 13.4 to more than 45%, compared to 9.4% in the general population [3]. They are also at greater risk of end-stage renal disease and myocardial infarction as a result of uncontrolled diabetes [4,5]. In the US territory of American Samoa, the most recently available national survey data reports a diabetes prevalence of 47.3% [95% CI: 44.0–50.7] among adults [6], far exceeding the prevalence in any other US state or territory^1^ and placing a catastrophic burden on a poorly resourced health system [7].

While several interventions targeting lifestyle modifications have shown efficacy in preventing or managing diabetes, few have specifically targeted ethnic minority groups. These interventions also tend to have poorer outcomes when tailoring for specific cultural practices, attitudes, and beliefs is absent [8,9]. PI culture, in particular, necessitates adaptation of interventions targeting lifestyle modification but to date, very few interventions have been developed specifically for this group [10,11]. The PI family structure (hierarchical, with multiple generations residing in the same home) and prioritization of social relationships alongside or even over individual health [12] present additional challenges for health care access and diabetes self-care, but they also present as yet unexplored opportunities. Likely because of these values, several family-centered weight loss interventions and one recent diabetes self-management intervention have been successful among PIs [13–15].

In other contexts, children and adolescents have been recognized as agents of familial health change and are often engaged as navigators of the health system [16–19]. Using the principles of reverse socialization, by which children alter their elders’ views and behaviors, the ‘Hip Hop Stroke’ program was able to increase stroke awareness and preventative behavior among economically disadvantaged African American and Hispanic parents and grandparents by delivering an educational intervention to their children [20]. Another study showed that without specified direction, children and adolescents (10–17 years of age) played important roles in parents’ diabetes management by monitoring dietary intake, helping with shopping and food preparation, encouraging and reminding parents to exercise, providing medication reminders, and assisting in glucose monitoring [21]. To our knowledge, however, there is no precedent for considering PI adolescents–who have clearly-defined roles in the PI family structure and a unique sense of responsibility for the wellbeing of their family members [22]–as possible agents of familial health behavior change.

We will extend the idea of using adolescents as agents of change to diabetes self-management among American Samoan adults. We will develop and test an adolescent-mediated intervention designed to improve the glycemic control and self-care practices of a paired, adult family member (parent or grandparent) with diagnosed diabetes. We will use experiential learning and facilitated discussion to increase adolescent diabetes knowledge, literacy, and numeracy; enhance their interpersonal communication and leadership skills; and equip them to assist their family member in navigating barriers to diabetes control and self-care. Adolescents will be the sole recipients of the active intervention and we will examine whether they can be effective conduits of diabetes knowledge and encourage familial behavior change by measuring changes in glycemic control and self-care behaviors of the paired, adult family member. While the focus of the intervention will be solely on improving their family member’s diabetes outcomes, we hypothesize that exposure to the intervention will also result in positive health behavior change among the adolescents themselves. This would be significant because of their inherent familial diabetes risk and would be a novel approach to simultaneously targeting management and prevention in the same family.

## Study objectives

Our research aims are:

1. To assess the preliminary efficacy of the adolescent-mediated intervention in improving adult diabetes outcomes (glycemic control, body mass index (BMI), blood pressure, waist circumference);
2. To assess the preliminary efficacy of the adolescent-mediated intervention in reducing adolescent risk factors for diabetes (glycemic control, BMI, blood pressure, waist circumference); and
3. To evaluate implementation outcomes (acceptability, feasibility, reach, fidelity) and factors influencing sustainability (program costs, likelihood of adoption).

## Materials and methods

**Fig 1:**
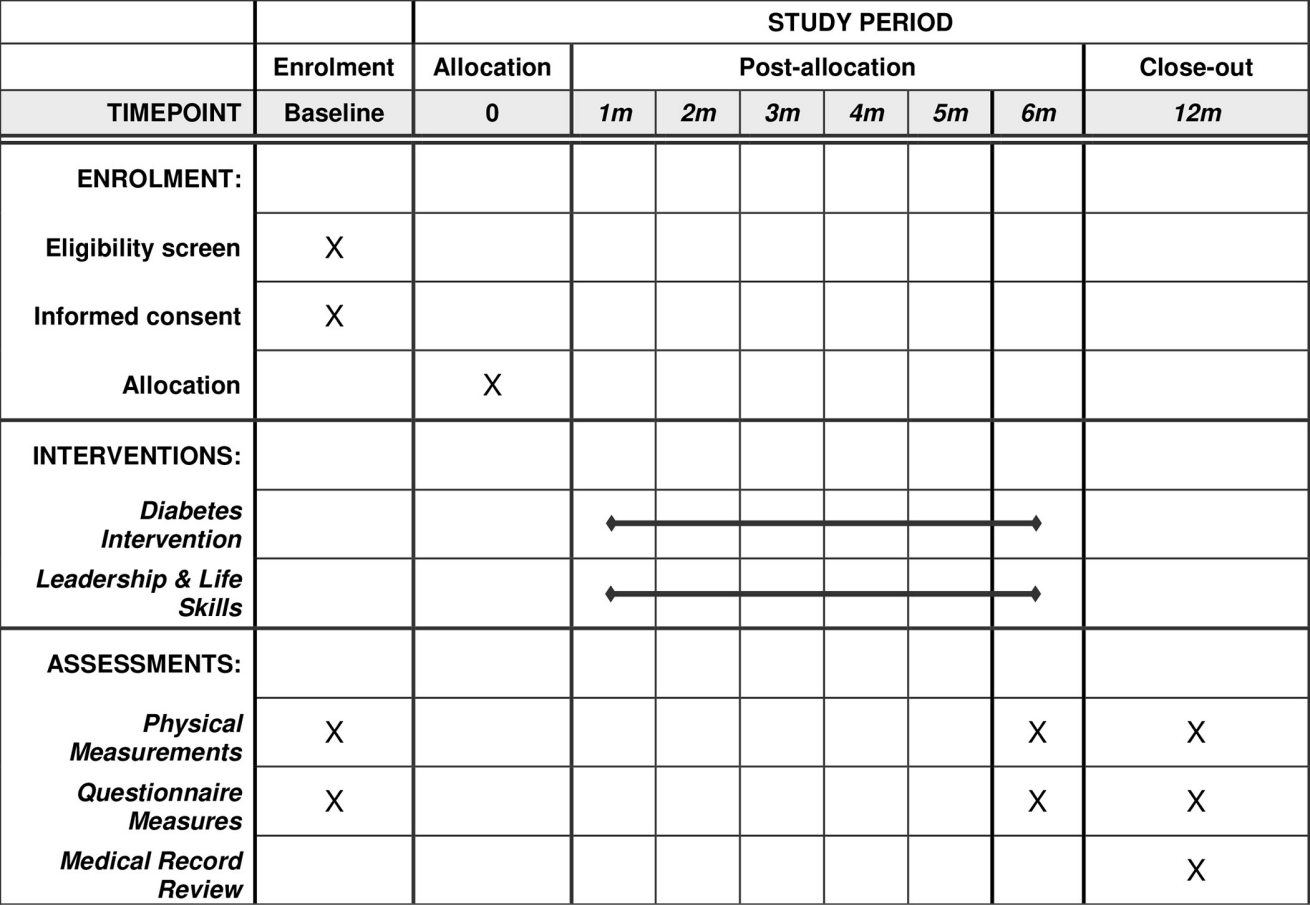
Schedule of enrolment, intervention, and assessments.

To test our hypothesis that adolescents can be effective conduits of diabetes knowledge and will support their paired family member in the adoption of self-care strategies, we will conduct a pilot, parallel group randomized controlled trial with n = 160 dyads (adolescents and a parent/grandparent with diabetes; Fig 1). Recruitment for the study will take place between June 2022 and January 2023. Adolescents from dyads randomized to the intervention group will participate in 12 group-based intervention sessions delivered over a period of six months. Adolescents randomized to the control group will be matched for contact and receive a non-diabetes focused leadership and life skills curriculum over the same six-month period. Aside from planned research assessments, we will have no contact with the adults in the dyad, who will proceed with their usual diabetes care. Research assessments will take place pre-randomization, after the active intervention phase (six months post-randomization), and at 12-months post-randomization, following a no-contact maintenance phase. We will collect implementation outcomes to examine feasibility, acceptability, cost, and sustainability. The study has been approved by the Institutional Review Boards at Yale University (Protocol #: 2000031325) and the American Samoa Department of Health. Protocol modifications will be reviewed and approved by both review boards.

### Setting

American Samoa is an unincorporated territory of the United States (US) and has a population of 55,197 (2020) [23], >90% of whom identify as Samoan and of PI ethnicity. American Samoans are US nationals, travel freely to and from the US, and serve in the US armed forces. Approximately 73% of the population lives at or below the federal poverty line (per capita Gross Domestic Product (GDP) is $11,535, compared to the US average of $63,544 [24]). American Samoa is eligible for federal block and categorical grant programs and derives a major portion of its annual budget (including its healthcare budget) from Department of the Interior Grant-in-Aid and Federal grants. The territory is considered a medically underserved population by HRSA [25]. There is one tertiary care facility (the Lyndon B Johnson Tropical Medical Center) close to the capital city of Pago Pago, and five federally qualified community health centers that are operated by the American Samoa Department of Health and provide primary care services.

### Participants

Eligible participants will be an adolescent (14–17 years) and a parent/legal guardian/grandparent who share the same household; both participants must be willing and consent to participating in the program together and be of Samoan ethnicity. The adult must have received a diagnosis of Type 2 Diabetes by a clinician at least 12 months prior to enrollment (to avoid immediate post-diagnosis behavior changes masking effects of the intervention), have glycated hemoglobin (HbA1c) ≥6.5%, and have been prescribed diabetes medication, whether they are currently taking medication or not. To be included, adolescents must be willing/able to participate in group intervention sessions. There will be no additional physical/ biochemical inclusion criteria, although given the population prevalence we expect the majority of participants (>65% of adolescents and ~90% of adults) to have overweight or obesity and up to 30% of adolescents will have prediabetes based on HbA1c [26].

Adult women/adolescent girls will be ineligible if they plan to become pregnant during the study period (and will be excluded from analyses if they become pregnant while enrolled based on anticipated pregnancy-related changes in key study outcomes). Both participants will be deemed ineligible if either are planning to leave American Samoa in the next 18 months or if they report any of the following: uncontrolled hypertension (systolic >180 mmHg or diastolic >105 mmHg), heart attack, stroke, or transient ischemic attack in the past year, treatment for cancer, chest pain or shortness of breath with minimal activity, chronic lung disease requiring home oxygen therapy, inability to read/speak Samoan and/or English, or contraindications to moderate physical activity. Adolescents will be excluded if they have overt diabetes (HbA1c ≥6.5%) based on testing during the screening process but will remain eligible if they have prediabetes (HbA1c >5.7%), since the intervention is likely to benefit them, particularly in the absence of any existing programs in American Samoa to support lifestyle intervention in adolescence. Any participant determined to have uncontrolled hypertension, or a new diagnosis of diabetes will be referred to the health system for follow up.

### Recruitment and randomization

Since adolescents will be the recipients of the intervention, they will be the major target of recruitment efforts. We will partner with Department of Education-run high schools and advertise the study to adolescents through assemblies, visits to classrooms in the age-range of interest, and posters placed around the schools. Interested participants will contact study staff by phone, text message, or Facebook, or deposit an expression of interest in designated boxes at the school. Adolescents will be encouraged to discuss their participation with the member of their family who they wish to participate with before contacting study staff. Adult participants will also be targeted through social media outreach, local media, referrals from healthcare providers, and through local parent-teacher associations. Project staff will visit interested adolescents and their family members in their home to explain the study, determine eligibility, and gain informed consent. Questionnaires, blood pressure, and HbA1c screening (using a point-of-care A1cNow device, PTS Diagnostics) will be used to determine eligibility.

If both members of the adolescent-adult pair are eligible based on the criteria described above and HbA1c screening, they will consent separately to participation (but both must agree). Adults will complete written informed consent forms; adolescents will give their written assent and will also be required to provide parental/legal guardian consent, if the adult they are participating with is not a biological parent or legal guardian. Adult participants will be asked to provide consent for the research team to review their medical record data maintained by the tertiary care center and primary care centers.

Following the completion of a pre-randomization research assessment (described below), dyads will be randomized to receive either the diabetes-focused intervention or a leadership and life skills curriculum. We will use block randomization, stratified by adolescent gender identity, with 4 total blocks (2 per intervention group) of 10 dyads each (5 per gender), to assign participants to their study groups. Group allocations will be made by the study biostatistician and concealed from study staff until participant assignment using opaque envelopes.

### Intervention

We will place adolescents into a group with nine others (n = 10 adolescents per group; gender-balanced). Individuals will attend all sessions with the same group. Groups will meet 12 times over six months with sessions lasting approximately 90 minutes each. Intervention sessions will be led by two American Samoan facilitators and delivered using a facilitated discussion approach.

Intervention content will focus on five key behaviors: (1) medication adherence, (2) primary care utilization, (3) physical activity, (4) mindful eating and consumption of less energy dense foods, and (5) stress reduction/sleep, all of which could be hypothesized to have a positive impact on our primary outcomes [27–30]. A prior nurse-community health worker led intervention study in American Samoa showed that increasing adherence to medication and improving primary care utilization can be effective in improving glycemic control [31]. Specific attention will be paid to these topics, by introducing them early in the curriculum (**Table 1**) and repeatedly reinforcing the importance of these behaviors. Adolescents will be encouraged to join their paired family member at health care appointments and will be equipped with skills necessary to facilitate their family member’s medication adherence.

**Table 1:** Intervention curriculum.

Session	Educational Focus
1	**What to expect; what is diabetes?** Explore risk factors, symptoms, and complications
2	**Diabetes treatment** Learn the importance of medication and treatment adherence
3	**Keeping track** Understand how to track blood sugar changes and identify and respond to emergency situations
4	**Making the best decisions** Explore ways to prioritize health
5	**Healthcare matters** Learn strategies for engaging with primary care and identify barriers to care
6	**All about food** Understand how diet contributes to diabetes risk and explore mindful eating
7	**Reaching activity goals** Learn how physical activity can promote health and manage diabetes risk
8	**Measuring health** Explore the ways that we can monitor our own health and wellbeing
9	**Sleep and Stress** Understand how to take a holistic approach to health
10	**Breaking down barriers** Strategize about how to solve common problems related to diabetes care and management
11	**Making sure medicines work** Review successes and challenges in medication adherence
12	**Maintaining healthy habits** Set goals for life after the intervention

Because the goal of this intervention is to empower and equip adolescents to support their family members in managing their diabetes, and the expectation is that adolescents will act as conduits for diabetes knowledge and agents of behavioral change, the intervention will build leadership and communication skills into each session to facilitate knowledge transfer between the adolescent and paired family member. Materials and activities are based on those used for a leadership and life skills camp successfully delivered to high-school and college athletes in American Samoa in 2015, 2017, and 2019 and accounts for Samoan-specific elements of the family environment and the unique roles and responsibilities of adolescents in this context. We will also use elements of the widely adopted Patient Navigator Training program, developed to train patient navigators to support interactions with cancer care [32]. Specifically, we will focus on activities from that curriculum that build foundational understanding of health behavior (grounded in dual-process and self-determination theory [33,34]), promote empathy, help adolescents provide information to their paired adult about their health condition in a way that they are able to understand, and educate adolescents to identify and address the structural and emotional barriers faced by their partner in managing their condition.

Finally, multiple studies—from the education literature to health interventions—have demonstrated that prior to adulthood, experiential learning is more effective for retention of information and behavior change [35]. Several behavioral interventions for diabetes have successfully incorporated cooking demonstrations and group physical activity [36–40]; we will include similar opportunities for experiential learning, with a focus on using local, healthy foods for cooking and exercise that can be tailored to suit all family members.

### Control condition

Adolescents randomized to the control group will meet for the same number of sessions but will receive only the leadership and life skills components of the intervention. Their sessions, which will last an hour, will be focused on building capacity for leadership and applying it to considering their future life and career goals and motivations. Facilitated discussion and experiential learning activities will be applied in the same way, but the focus will not be on health generally, or diabetes explicitly. Written educational materials based on the diabetes intervention curriculum will be provided to the dyads randomized to the control condition at the end of their participation in the study.

### Data collection

Participants (adolescent and adult) will complete assessments prior to randomization (baseline), after the active intervention phase (6-months post-randomization), and after a six-month, non-active maintenance phase (12-months post-randomization). All assessments will be conducted by a trained research assistant blinded to group assignment and participants will be invited to complete assessments regardless of intervention adherence.

### Physical measurements

The primary study outcomes are adult glycemic control (HbA1c) and cardiovascular risk factors (BMI, waist circumference, blood pressure). We will measure HbA1c using a point-of-care device (A1cNow, PTS Diagnostics). The measure collected during the recruitment and screening process (to determine eligibility) will be used as the baseline measure. Body weight and height will be measured using a SECA portable stadiometer and Tanita HD 351 digital scale, respectively, and will be used to calculate BMI. Waist circumference, a proxy for visceral adiposity, will be measured using a cloth measuring tape. Blood pressure will be measured in triplicate on the non-dominant arm, using an automated sphygmomanometer (Omron HEM 907XL) at five-minute intervals. The same outcomes will be collected among adolescents (secondary study outcomes). If at any point in the trial either participant develops uncontrolled hypertension or the adolescent develops diabetes they will be referred for medical care.

### Questionnaire measures and medical record review

Since social and environmental factors have a considerable impact on the potential effectiveness of health interventions, we will collect data from participating families to assist in exploring why the program is or is not effective (covariates) and by what behavioral pathways the intervention may have exerted its effect (process). Because the intervention approach uses adolescents as agents of change, we will also attempt to measure communication between the adolescent-adult pair, and what information the adult recalls receiving from the adolescent (process). We will also record participant age, self-reported gender, and biological relationship between the pair (parent and child/grandparent and child) since these characteristics may influence outcomes. Questionnaire measures that will be used in the study are described in **Table 2**.

**Table 2:** Questionnaire measures.

Questionnaire	Description	Adolescent			Adult		
		BL	6m	12m	BL	6m	12m
*Covariates*							
Demographic Characteristics	Participant age, gender, relationship between the pair (parent, grandparent)	X			X		
Household Characteristics	Household size, socio-economic position, food security				X		
Health Literacy	Adults: Brief Health Literacy Screening Tool (BRIEF) [41]; Adolescents: Health Literacy for School-Aged Children (HLSAC) [42].	X	X	X	X	X	X
Health Interview	Other diagnoses, medication use (for diabetes and other conditions), diabetes self-monitoring, healthcare utilization, self-reported health				X	X	X
Perceived Risk of Diabetes	Risk of developing diabetes in the next five years, in their lifetime, and perceived disease severity [43–45]	X	X	X			
*Process Measures*							
Medication Adherence	Modified version of the Hill Bone high blood pressure therapy scale [46]				X	X	X
Diabetes Empowerment	Diabetes-related psychosocial self-efficacy; Diabetes Empowerment Scale-Short Form (DES-SF) [47]				X	X	X
Diabetes-related Distress	Diabetes-related psychosocial distress; Diabetes Distress Scale (DDS-17) [48]				X	X	X
Dietary Intake	30-day recall of dietary behaviors: National Health and Nutrition Examination Survey Dietary Screener Questionnaire (DSQ) [49]	X	X	X	X	X	X
Physical Activity	7-day recall of physical activity behaviors’ Global Physical Activity Questionnaire (GPAQ) [50]	X	X	X	X	X	X
Stress and Depression	General stress and depressive symptoms: Cohen’s Perceived Stress Scale (PSS) [51], Patient Health Questionnaire (PHQ-9) [52]	X	X	X	X	X	X
Cigarette and Alcohol Consumption	Current use of tobacco (manufactured, local, e-cigarettes) and alcohol	X	X	X	X	X	X
Sleep	Average nightly sleep, daytime sleepiness, sleep quality, insomnia	X	X	X	X	X	X
Health Locus of Control	Internality, powerful others externality, chance externality; Multidimensional Health Locus of Control Scale (MHLC) [53]				X	X	X
Relationships and Communication	Quality of the relationship between adolescent and adult participant; Network of Relationships Inventory [54]	X	X	X	X	X	X
Instrumental and Emotional Support	Perceived degree of instrumental and emotional support provided by adolescent				X	X	X
*Self-Reported Diabetes Outcomes*							
Diabetes Symptoms	Diabetes symptom burden; Diabetes Symptom Checklist (DSC-R) [55]				X	X	X

BL = Baseline (pre-randomization); 6m = 6 months (post-intervention); 12m = 12 months (post-maintenance phase).

Electronic medical records will be reviewed to document the health care utilization of the adult participants (primary care, emergency care, inpatient stays, preventative medicine [dental, ophthalmic, foot clinic, mental health services] and preventative screening), prescribed medication (diabetes-specific, i.e., prescriptions and refills) and access to other standard care recommendations such as flu and COVID-19 vaccination [56]).

#### Qualitative data collection

We will conduct semi-structured interviews with 20 pairs of participants randomized to receive the diabetes intervention–specifically, with the 10 dyads who experience most “success” in the trial (in terms of adult HbA1c) and the 10 least successful. Interviewing the pair together, we will focus on what aspects of their communication and behavior changed over the course of the intervention and their beliefs about why they had more or less success.

### Participant retention

To maximize retention of participants for the duration of the proposed study we will employ a number of strategies: (1) we will obtain detailed primary and secondary contact information from participants and will update/confirm these contact details at each assessment visit; (2) we will schedule study visits at convenient times and places, (3) we will use several means to remind participants of intervention visits and follow-up assessments, including appointment cards, telephone calls, and text messages; (5) we will provide regular study updates and opportunities for engagement through our dedicated Facebook page (@YaleOlaga); and (6) we will offer small incentives for participation in the research assessments ($20 for each assessment, $60 total). We will monitor retention patterns continually using a comprehensive participant tracking system to record attempted contacts and missed and attended appointments and will review this information regularly.

If either an adult or an adolescent is permanently lost to follow up or must be excluded (for example, moves out of American Samoa, becomes pregnant, or develops a medical condition that precludes continued participation, among others) the remaining member of the pair will be allowed to continue in the intervention and complete the assessment visits as planned. If an adolescent is lost to follow up post-randomization after receiving even some of the intervention, there may be measurable effects in the adult family member (although the circumstances of adolescent loss to follow up will be reviewed before including the adult data in data analysis). If the adult participant is lost to follow up, we will still be able to address secondary outcomes (adolescent risk factor reduction).

Participants who withdraw from the study will be asked whether their existing data may be included in future analyses. If they wish for their data to be removed, it will be removed permanently from the dataset and all further analysis. Because one of the primary goals of this study is to establish intervention acceptability, we will ask participants who withdraw to share their reasons for withdrawal if they are willing. We will still routinely invite participants who withdraw to any events that communicate study findings.

### Protection of human subjects

Risks associated with participation in this study are considered to be minimal. We will ask a healthy, community sample of adolescents randomized to the intervention to transmit knowledge and to provide support to a family member living with type 2 diabetes. We will have no contact with the adult participants during the intervention aside from their research visits and they will continue with their usual diabetes care, under the supervision of a licensed health practitioner. Given that the intervention and control sessions will happen in groups, the primary risk is loss of confidentiality. We will mitigate this by reminding participants about the importance of maintaining the confidence of other group members prior to participation. We will also employ a number of safeguards to protect data collected, including assigning unique ID numbers to participants. If a participant feels uncomfortable discussing any particular topic, they will be informed that they have the right to leave a conversation or to leave a questionnaire measure unanswered. The physical measures that will be collected (namely blood pressure and HbA1c) may present additional risks to patients in the form of temporary discomfort/bruising. Study staff will be fully trained in procedures necessary to collect biochemical and physical outcomes. Since this trial is considered to have minimal risks for participants, study monitoring will be the responsibility of the principal investigator (NLH). Unanticipated problems, protocol deviations, and adverse events will be reported to the overseeing institutional review boards within 48 hours of the investigators becoming aware of the event.

### Intervention evaluation

We will integrate evaluation activities throughout the implementation of the intervention to capture outcomes critical to understanding the potential for adoption into practice and future scaling in American Samoa and more broadly. Using best practices from implementation science [57,58] we will document acceptability, reach and adoption, feasibility, fidelity, cost, and potential for sustainability and scale-up. Activities to be used and the timing of data collection are described in **Table 3**.

**Table 3:** Intervention evaluation activities.

Outcome	Evaluation Measures	Participants/Timeline
**Acceptability**	Acceptability [59] will be determined based on *participant satisfaction* which we will measure using: • Structured questionnaires: perceived participation burden, opportunity costs, experience of participation, attitude toward intervention and associated activities • Focus Group Discussions (FGDs): FGDs will be conducted with adolescent and adult participants separately. Topics: similar to the questionnaire measures • Stakeholder interviews: (n = 10–15; research staff responsible for intervention delivery, health system leadership) experiences of intervention implementation, burden of delivery, feedback from participants • Brief interviews with participants who withdraw, primary reasons for discontinuation	Intervention participants and stakeholders; after completion of the active intervention phase (6m post-randomization) Study non-completers (upon notification of withdrawal)
**Reach and Adoption**	Measured using elements of the RE-AIM framework [60] reach and adoption will be estimated using: • Recruitment metrics: number of potential participants included/excluded, % who participate, characteristics of participants vs. general population • FGDs and stakeholder interviews: study staff experiences of recruitment, stakeholder perceptions of potential adoption among community organizations	Stakeholders; upon completion of recruitment activities and after implementation is complete
**Feasibility**	Feasibility will be determined based on *recruitment*, *enrollment*, and *retention* rates as well as *adherence* and *engagement* with intervention activities • Retention and adherence metrics and drop-out interviews: % of participants who do not complete the study; number of sessions attended • Brief interviews with participants who withdraw, barriers to participation • Completion of measurement tools: % of missing data • Research staff interviews: challenges with program delivery or measurement of outcomes	Study non-completers (upon notification of withdrawal); Health educators (after implementation is complete)
**Fidelity**	Fidelity to the intervention curriculum (for both intervention and control groups) measured using: • Observations/audio recordings of sessions: adherence to planned content, completion of planned experiential learning activities, quality of delivery (interventionist enthusiasm, communication style, and confidence) • Fidelity checklists: intervention dose (duration of study sessions, interaction with participants outside of formal sessions) • Structured questionnaires: adolescent report of achieving learning outcomes (short (2–3 question) surveys will be completed after each group session)	Study PI (review of session content) Interventionists (after each session) Adolescents (after each session)
**Cost**	Program costs will be evaluated using a *micro-costing approach* [61] to generate estimated per-participant costs: • Structured questionnaires: participants (adolescent and adult) will estimate time and resources spent participating (travel, food costs, etc.), medical costs incurred • Medical records: participant medical costs • Stakeholder interviews/questionnaires: health system accounting departments will provide estimates of direct medical costs and non-medical costs, personnel costs (salaries), intervention materials, facility-level overhead costs	Intervention Participants; after the active intervention phase Stakeholders; after implementation is complete
**Sustainability and Potential to Scale**	Assessment of potential for scale-up and sustainability will be guided by WHO steps for developing a scaling-up strategy (steps 3 & 4) [62] and will focus on *environment* and *human capacity*. • Stakeholder interviews: identification of key stakeholders for scale-up, political/policy connections needed, related initiatives that could be leveraged, likely barriers to scaling, leadership and advocacy potential within the health system, experience with successful scaling of other programs, stability of human resources, motivation of health system leadership to sustain the program	Stakeholders; after implementation is complete

### Analytic approach and statistical considerations

#### Data management

Questionnaire data, medical record data, and physical measurements will be captured by research assistants using REDCap Software. Database downloads and backups will be stored on Yale Secure BOX. Access to the dataset will be restricted to those with IRB approval and appropriate training. Data will be checked for out-of-range or inconsistent values before use in analyses.

#### Trial outcomes

Using an intent-to-treat approach, we will compare group differences in primary and secondary outcomes post-active intervention and following the maintenance phase. Generalized estimating equations (GEE) with the robust “sandwich” variance estimator accounting for the within-cluster correlation will be used to model outcomes post-active intervention and then separately following the maintenance phase. For continuous outcomes (e.g., HbA1c) we will use these models to examine differences in (1) the mean outcome and (2) the rate-of-change of the outcome between the intervention and control conditions. Each of the outcomes will be modeled individually. If significant differences between groups are identified post-intervention/maintenance, we will conduct subgroup analyses to understand the process underlying those differences using GEE. Prior to analysis, the demographic characteristics and baseline measures will be compared between the intervention and control conditions. Any measures demonstrating evidence of difference between intervention and control, in addition to any relevant participant characteristics, such as age and gender, will be included in all analyses of post-active intervention and maintenance outcomes. Since this is a pilot study to provide estimates for a larger trial, there will be no adjustment for multiple comparisons.

While this project will not be adequately powered to test for these interactions, we will perform exploratory analyses of gender-specific effects as we expect that intervention efficacy may vary based on (a) the adolescent’s gender, (b) the adult’s gender, and (c) the gender match between the pair. To explore this, we will estimate the statistical interactions between these variables and intervention/control group assignment. We will compare estimated outcomes from the statistical models to see if there is, at a suggestive and qualitative level, any differences in intervention efficacy that could inform future intervention design or be further examined in larger trials.

#### Intervention evaluation

Quantitative data will be summarized descriptively, with paired samples t-tests and generalized linear models used for between group comparisons as appropriate. Focus groups (FGDs) with study participants and semi-structured interviews (SSIs) with stakeholders (clinicians, DOH representatives, diabetes educators, community leaders) will be used to evaluate several of the implementation outcomes described in **Table 3**. We will complete FGDs with all adolescents (in their original intervention and control groups, as part of the final group meeting). We will also conduct four FGDs with adults from dyads randomized to the intervention. SSIs with stakeholders will be one-on-one, since in previous research we have found scheduling and employment hierarchies challenging to navigate in an FGD setting. Both FGDs and SSIs will use a combination of open-ended questions and directed probes to explore concepts of interest and will be conducted in English (since >95% of the population are fluent). Transcripts will be entered into NVivo analysis software and coded using topics developed from the FGD and SSI agendas (deductive codes) and content from the transcripts (inductive codes). Content analysis will be used to identify broad themes and patterns related to implementation outcomes and germane to future iterations of the intervention, answering both our *a priori* research questions (*how acceptable and feasible is this intervention*? *What is the potential for sustainability and scale-up*?) and identify new themes that emerge from the data.

## Discussion

Despite being among the fastest growing US population groups (having increased by >40% between 2000 and 2010) [63–65], PIs are underrepresented in health research and innovations in diabetes care have been slow to reach them. Novel treatment and prevention strategies, specifically targeted to this group, are critically needed to reduce health disparities. Our proposed work is novel and innovative in that this will be the first study to engage adolescents as agents of change in a diabetes intervention. Social support, especially family support, in the form of education, emotional support, and aid in decision making, has been demonstrated to increase the effectiveness and maintenance of diabetes self-management strategies and to improve clinical outcomes [66]. But, while precedent exists for using children/adolescents as conduits of health knowledge for other conditions or parents as agents of childhood/adolescent behavior change, our formalized approach to equipping adolescents with the skills to support an adult family member’s diabetes self-management represents a novel paradigm shift. In addition, we will target management and prevention of diabetes simultaneously by enrolling adolescents at risk of diabetes based on ethnicity and family-history and their family members, who are already managing the condition.

While many family-based interventions have been conducted [66], no interventions explicitly attempt to target behavior change beyond the patient with diabetes. In a 2016 systematic review of 26 family-based interventions to improve diabetes outcomes among adults, engagement of family members varied widely [66]: only two interventions provided systematic training for family members to play a supportive role [67,68], and only four measured outcomes among family members [69–71] In the mainland US, 20% of the US population live in multigenerational households [72], with the highest proportions among the Asian, PI, and African American communities–all of whom could benefit from diabetes intervention should this pilot study be effective.

Intervention success, from an implementation perspective will be judged based on perceived feasibility, acceptability, cost, and potential for scale-up. Among the most important benchmarks for success will be participant report that they would choose to participate again, health educator report that they believe the intervention was effective, and willingness of health system leaders to invest in the next steps toward scale-up. The intervention will be judged to be efficacious if it impacts adult glycemic control, cardiovascular risk factors, and/or self-care behaviors, since all are likely to have a positive impact on diabetes progression.

Results of this study will be disseminated first to participants and stakeholders in American Samoa. Knowledge translation activities will include academic publications and conference presentations, community and government reports, community discussions, and media/social media activities. Results will also be disseminated through ClinicalTrials.gov.

There are several potential limitations to this study protocol. First, the study was not designed as a fully powered trial. As such, we may have limited statistical power to examine some outcomes. Second, while we will attempt to recruit equal numbers of male and female adolescents, we will not attempt to control the gender of the adult they choose to participate with. The gender match of the pair (same or opposite) may affect communication [73,74] and intervention effectiveness. As described, the sample size may be too small to examine effects quantitatively, but we will attempt to explore this qualitatively in the proposed paired semi-structured interviews and will be purposeful about attempting to get representation from both types of pairs in those interviews. Third, contamination may be a challenge. The close-knit social structure and geography mean that participants assigned to opposite groups may interact, likely at church (as was the case in other intervention studies in this setting [27]) or other social environments. We expect the impact of any contamination on the primary outcomes (adult health outcomes and behaviors) to be minimal because of the mode of intervention delivery, but we will attempt to measure contamination and control for it in analyses. Finally, we recognize concerns among medical providers and professional organizations about the undue burden placed on children/adolescents who serve as health care navigators for their parents/grandparents (a particular issue among some immigrant populations in the US [75]). One of the primary reasons for integrating leadership and communication skills throughout the intervention will be to mitigate any feelings of burden by the adolescent and we will address this explicitly in focus groups and semi-structured interviews to provide evidence on this topic that can be used by decision makers in considering scaling in other settings.

In conclusion, this study will provide important insight into the potential for Samoan adolescents to act as agents of familial health behavior change. Successful completion of our aims and proof of efficacy would produce a scalable program with high potential for replication in other similar, low-resource, family-centered, ethnic minority groups across the US who are the ideal beneficiaries of innovations to reduce chronic disease risk and eliminate health disparities.

## Supporting information

S1 Checklist SPIRIT 2013 checklist: Recommended items to address in a clinical trial protocol and related documents*. (DOC)

S1 File (DOCX)

## Abbreviations

BMI: Body Mass Index
CI: Confidence Interval
FGD: Focus Group Discussion
GDP: Gross Domestic Product
GEE: Generalized Estimating Equation
HbA1c: Glycated Hemoglobin
PI: Pacific Islander
US: United States
