# Supplementary material for: Protocol: Implementation and evaluation of an adolescent-mediated intervention to improve glycemic control and diabetes self-management among Samoan adults
Source: PLoS One. 2023 Feb 16;18(2):e0279084. doi: 10.1371/journal.pone.0279084 (PMC9934313; doi:10.1371/journal.pone.0279084)
Supplement: S1 File — (DOCX) [file pone.0279084.s002.docx]

**Family Diabetes Intervention – American Samoa**

Protocol Number

2000031325

Protocol Version

February 10, 2022

2.0

# Synopsis

| PurposeThe number of individuals diagnosed with Type 2 Diabetes in the United States (US) has more than doubled since 2000 to over 30 million, with an additional 84.1 million living with prediabetes. Although several preventative interventions have demonstrated efficacy, they are not adequately reaching the ethnic minority groups on whom a disproportionate burden of diabetes is falling. One minority group at particular risk is Pacific Islanders, who are underrepresented in health research and who innovations in diabetes care have been slow to reach. This randomized controlled trial will investigate whether adolescents can be engaged as agents of change within their family to improve the diabetes-related health outcomes of their family members (parents, legal guardians, or grandparents). We will test the intervention in American Samoa, where diabetes prevalence is among the worst in the US. Successful completion of our aims and proof of efficacy would produce a scalable program with high potential for replication in other similar, low-resource, family-centered, ethnic minority groups across the US who are the ideal beneficiaries of innovations to reduce chronic disease risk and eliminate health disparities. |
| --- |
| Primary Objective The primary objective of this study is to examine the efficacy of an adolescent-focused diabetes intervention (delivered solely to adolescents) in improving the diabetes outcomes of a paired family member (parent/legal guardian, grandparent) with diabetes. Outcomes will include the **family member’s** glycemic control (HbA1c), body mass index (BMI) and waist circumference. |
| Secondary Objectives The secondary objectives of the study are as follows:   1. To assess the preliminary efficacy of the program in reducing **adolescent** risk factors for diabetes (HbA1c, BMI, blood pressure, waist circumference) 2. To evaluate implementation outcomes (acceptability, feasibility, reach, fidelity) and factors influencing sustainability (program costs, likelihood of adoption) |
| Study DesignWe will recruit and randomize n=160 dyads (an adolescent (without diabetes) and a parent or grandparent diagnosed with diabetes). Dyads will be randomized 1:1 to the intervention or a control condition. Adolescents from dyads randomized to the intervention group will participate in 12 group-based intervention sessions delivered over a period of six months. Adolescents randomized to the control group will be matched for contact and receive a non-diabetes focused leadership and life skills curriculum over the same six-month period. Aside from planned research assessments we will have no contact with the adults in the dyad, who will proceed with their usual diabetes care. To test our hypothesis that adolescents receiving the intervention will be effective conduits of diabetes knowledge and will support their paired adult in the adoption of self-care strategies, our primary efficacy outcomes will be adult glycemic control and cardiovascular risk factors (BMI, blood pressure, waist circumference). Secondarily, since we believe that exposure to the intervention may encourage positive behavior change in the adolescent themselves, we will measure adolescent glycemic control, cardiovascular risk factors, and risk behaviors. Outcomes will be measured at baseline, after the active intervention phase (six months post-randomization) and at 12-months post-randomization, to examine maintenance of intervention effects in the absence of contact. We will collect implementation outcomes to examine feasibility, acceptability, cost and sustainability. |
| Study Date Range and Duration The study will take place over a period of **four years** (December 2021 to September 2025). Recruitment is anticipated to begin in February 2022 and the final study assessments will take place in March 2024. The remaining study period will be used for analyses and dissemination of study findings. |
| Number of Study Sites There will be **four study sites**:   1. Yale University (School of Public Health, School of Medicine) 2. American Samoa Community College (where active data collection will be based) 3. University of Pittsburgh (analysis of de-identified data) 4. The Miriam Hospital, Rhode Island (analysis of qualitative evaluation data. The PI at the Miriam Hospital will have contact with participants during interviews) |
| Primary Outcome Variables Primary outcome variables will be measured among **family members** (parents, legal guardians, grandparents) of the adolescents taking part in the intervention. Outcomes will be:   1. Glycated hemoglobin (HbA1c); a measure of glycemic control over an ~3 month period. HbA1c is expected to improve if the intervention is efficacious since the intervention targets diabetes education, medication adherence, and lifestyle change. 2. Body Mass Index (BMI); a measure of weight proportional to height. BMI is expected to improve if the intervention is efficacious because of the focus of the intervention on lifestyle change (diet and physical activity). 3. Waist circumference; a measure of central body fatness. Waist circumference is expected to improve if the intervention is efficacious because of the focus of the intervention on lifestyle change (diet and physical activity). |
| Secondary and Exploratory Outcome Variables (if applicable) Secondary outcome variables will be measured among the **adolescents** receiving the intervention. Outcomes will be:   1. Glycated hemoglobin (HbA1c); a measure of glycemic control over an ~3 month period. HbA1c is expected to improve if the intervention is efficacious since the intervention targets diabetes education, medication adherence, and lifestyle change. 2. Body Mass Index (BMI); a measure of weight proportional to height. BMI is expected to improve if the intervention is efficacious because of the focus of the intervention on lifestyle change (diet and physical activity). 3. Waist circumference; a measure of central body fatness. Waist circumference is expected to improve if the intervention is efficacious because of the focus of the intervention on lifestyle change (diet and physical activity).   We will collect a number of process measures to determine how the intervention may impact these outcomes. These will include: social/emotional relationship between the adolescent and family member taking part, self-care behaviors among both adolescent and adult (diet, physical activity, stress, sleep, smoking, alcohol use), diabetes literacy among adolescents and adults, and medication adherence and healthcare utilization among family members only. Process measures will be collected with both quantitative and qualitative approaches.  We will also collect information about intervention acceptability, feasibility, reach, fidelity, and factors influencing sustainability. These data will be collected from intervention participants as well as local stakeholders using quantitative and qualitative approaches. |
| **Study Population**  The study will enroll n=160 dyads (n=320 total individuals). Each dyad will comprise an adolescent (14-17 years; healthy, without diabetes) and a paired family member (parent, legal guardian, grandparents with diabetes). Recruitment efforts will target adolescents and will be focused on four Department of Education-run high schools.  We will also enroll up to 25 stakeholders (study staff, health care providers, health leadership, and other relevant community members) to participate in evaluation activities. |
| Number of Participants The study will enroll **n=160 dyads (n=320 total individuals).** Reflecting the exploratory nature of the study, the sample size was determined based on both feasibility and statistical power. In an intent-to-treat analysis we will have approximately 40% power to detect a standardized between-group difference of 0.3 (i.e. the mean outcome differs between intervention and control by 0.3 standard deviations or approximately 0.68%) and >80% power to detect a standardized between-group difference of 0.5 (approximately 1.13%). We will have >90% power to detect changes in categorical outcomes (% participants meeting recommended dietary intake, for example).  Up to 25 stakeholders will participate in program evaluation activities including semi-structured interviews. |
| Study Schedule Adolescents enrolled in the study will have 15-16 interactions with the study as follows: the screening/consent visit (60-90 minutes, which may be divided into two visits depending on participant preference), research assessments at 6- and 12-months post-randomization (60 minutes each), and 12 intervention sessions (90 minutes each). Paired family members will have three interactions with the study – they will participate in the screening/consent visit (60-90 minutes, with the adolescent) and research assessments at 6- and 12-months post-randomization (60 minutes each).  Stakeholders participating in the evaluation interviews will meet with the research staff once and the interview will take approximately 45-75 minutes to complete. |

**Protocol Revision History**

| **Version Date** | **Summary of Substantial Changes** |
| --- | --- |
| December 8, 2021 | Initial protocol draft |
|  |  |
|  |  |

# Statement of Compliance

This document is a protocol for a human research study. The purpose of this protocol is to ensure that this study is to be conducted according to the Common Rule at 45CFR46 (human subjects) and other applicable government regulations and Institutional research policies and procedures.

# Abbreviations

| **Abbreviation** | **Explanation** |
| --- | --- |
| BMI | Body Mass Index; a measure of weight proportional to height, expressed as kg/m^2^ |
| CAB | Community Advisory Board |
| HbA1c | Glycated hemoglobin; a measure of long-term diabetes control, expressed as % |
| PI | Pacific Islanders |

# Glossary of Terms

| **Glossary** | **Explanation** |
| --- | --- |
| **Body Mass Index (BMI)** | A measure of weight proportional to height, expressed as kg/m^2^ |
| **Glycated Hemoglobin (HbA1c)** | A measure of long-term diabetes control, expressed as % |

Table of Contents

Synopsis 2

Purpose 2

Primary Objective 2

Secondary Objectives 2

Study Design 2

Study Date Range and Duration 3

Number of Study Sites 3

Primary Outcome Variables 3

Secondary and Exploratory Outcome Variables (if applicable) 3

Number of Participants 4

Study Schedule 4

Statement of Compliance 7

Abbreviations 8

Glossary of Terms 9

1 Background/Literature Review 15

1.1 Background 15

1.2 Prior Experience (if applicable) 17

2 Rationale/Significance 19

2.1 Rationale and Study Significance 19

2.2 Purpose of Study/Potential Impact 19

2.3 Potential Risks and Benefits 20

2.3.1 Potential Risks 20

2.3.2 Potential Benefits 20

3 Study Purpose and Objectives 21

3.1 Hypotheses 21

3.2 Primary Objective 21

3.3 Secondary Objective (if applicable) 21

4 Study Design 22

4.1.1 General Design Description 22

4.1.2 Study Date Range and Duration 22

4.1.3 Number of Study Sites 22

4.2 Outcome Variables 22

4.2.1 Primary Outcome Variables 22

4.2.2 Secondary and Exploratory Outcome Variables (if applicable) 23

4.3 Study Population 23

4.3.1 Number of Participants 23

4.3.2 Eligibility Criteria/Vulnerable Populations 24

5.1 Study Procedures 26

5.1.1 Data Collection 26

5.1.2 Adverse Events Definition and Reporting 31

5.2 Study Schedule 32

5.3 Informed Consent 32

5.3.1 Screening (if applicable) 33

5.3.2 Recruitment, Enrollment and Retention (if applicable) 34

5.3.3 Study Visits 36

5.4 Statistical Method 38

5.4.1 Statistical Design 38

5.4.2 Sample Size Considerations 39

5.4.3 Planned Analyses 39

5.4.4 Analysis of Subject Characteristics (if applicable) 40

5.4.5 Interim Analysis (if applicable) 40

5.4.6 Handling of Missing Data 40

6 Trial Administration 41

6.1 Ethical Considerations: Informed Consent/Assent and HIPAA Authorization 41

6.2 Institutional Review Board (IRB) Review 41

6.3 Subject Confidentiality 42

6.4 Deviations/Unanticipated Problems 43

6.5 Data Quality Assurance 44

6.6 Study Records 44

6.7 Access to Source 44

6.8 Data or Specimen Storage/Security 44

6.9 Retention of Records 45

6.10 Study Monitoring 45

6.11 Study Modification 45

6.12 Study Completion 45

6.13 Funding Source 45

6.14 Conflict of Interest Policy 46

6.15 Publication Plan 46

Appendices 47

List of Tables 48

Bibliography 49

# Background/Literature Review

## Background

## Using a randomized controlled trial design the primary objective of this study is to examine the efficacy of an adolescent-focused diabetes intervention (delivered solely to adolescents) in improving the diabetes outcomes of a paired family member (parent/legal guardian, grandparent) with diabetes. The trial will take place in American Samoa.

In 2015, 9.4% of the US population had diabetes - an estimated 30.3 million people^1^. In spite of significant medical advances and prevention efforts the burden of type 2 diabetes is expected to increase by more than 54% to exceed 59.4 million Americans between 2015 and 2030^2^, costing the US economy $327 billion in direct medical costs and lost productivity annually^3^. This burden falls disparately on racial and ethnic minority groups, who have a higher prevalence of diabetes, a greater burden of disease, and also experience a higher rate of complications^4^. Among them, Native Hawaiians and Pacific Islanders (NHPIs) are the most affected; prevalence estimates among NHPIs in the contiguous US range from 12.0-19.1%^5^. In the US territory of American Samoa, the most recently available national survey data reports a prevalence of 47.3% [95% CI: 44.0-50.7] among adults^6^, far exceeding prevalence in any other US state or territory^1^ and placing a catastrophic burden on a poorly resourced health system^7^. Like many other minority groups, American Samoans and PIs across the US, face significant barriers to healthcare access: they have lower income, have poorer health literacy, and often face linguistic barriers and a lack of culturally appropriate care^5^. Despite being among the fastest growing US population groups (having increased by >40% between 2000 and 2010)^8-10^, PIs are underrepresented in health research and innovations in diabetes care have been slow to reach them. Novel treatment and prevention strategies, specifically targeted to this group, are critically needed to reduce health disparities.

While several interventions targeting lifestyle modifications have shown efficacy in preventing or managing diabetes, few have specifically targeted ethnic minority groups and they tend to have poorer outcomes where tailoring for specific cultural practices, attitudes, and beliefs is absent^11,12^. Conversely, a recent systematic review showed that when diabetes interventions were targeted toward ethnic minority groups specifically, and tailored appropriately, most (74%) had a successful impact on glycemic control, fasting glucose, and/or weight loss^13^. PI culture, in particular, necessitates adaptation of interventions targeting lifestyle modification but to date, very few interventions have been developed specifically for this group^14,15^. Even in the face of significant modernization or migration to the US mainland, conceptions of health among PIs differ from the ‘Western’ definition^16^. Pacific ideas of health are closely linked to cultural identity^17^ and include holism, spirituality, and obligations centered around the extended family and communalism. Harmonious social relationships – particularly among family members – are an important component of health, such that “one is not considered healthy unless their social relationships are in good order”^16,18,19^. Likely because of these values, several family centered weight loss interventions and one recent diabetes self-management intervention have been successful among PIs^20,21,22^. Our proposed intervention is responsive to Samoan culture and by incorporating engaging family members in diabetes care, we are more likely to be successful in achieving the behavior change necessary to improve diabetes outcomes. This approach is likely to be relevant and externally generalizable to many other family-centered, ethnic minority groups.

Children have long been recognized as agents of familial health change and are often engaged as navigators of the health system where there are language barriers between patient and provider^23-26^. Recently, using the principles of reverse socialization, by which children alter their elders’ views and behaviors, the ‘Hip Hop Stroke’ program was able to increase stroke awareness and preventative behavior among economically disadvantaged African American and Hispanic parents and grandparents by delivering an educational intervention to their children^27^. A recent study showed that without specified direction, children and adolescents (10-17 years of age) play important roles in parents’ diabetes management by monitoring dietary intake, helping with shopping and food preparation, encouraging and reminding parents to exercise, providing medication reminders and assisting in glucose monitoring^28^. We are proposing to build on this, by formalizing the role of adolescents and delivering the intervention to them in the hope that they can serve as conduits for diabetes knowledge and supporters of behavior change. This is likely to be effective since adolescents – specifically in the age range we are proposing (14-17 years) - have a unique role in the Samoan family structure; they are expected to show respect, service, and deference to family, increasing their responsibility within the family unit as they move out of childhood. These qualities are then rewarded with increasing autonomy and recognition as a rite of passage into adulthood^29^. We are cognizant of the critiques levied at interventions that have attempted to engage children or adolescents as caregivers, health system navigators, or agents of familial change^30^. A recent review argued, however, that the benefits of leading health promotion efforts in the family outweigh any potential structural (time) or emotional burden^26^. The authors describe how in American households this role allows children to strengthen their cultural identity and resiliency, gain reciprocal support from family members, build self-esteem and self-efficacy, and feel a sense of belonging^26^. Additionally, young caregivers have reported an increased sense of self-worth, pride, accomplishment, and development of new life skills^30^. Ensuring these potential benefits led to the inclusion of leadership, communication, and life skills in our intervention approach.

## Targeting multiple members of the same family, and specifically multiple generations sharing the same home, is important because of the role that family can play in health and also because having a family member with diabetes is one of the most salient risk factors for diabetes in youth^31,32^. Type 2 diabetes prevalence among adolescents in the US increased by more than 30% between 2001 and 2012; if this rate of increase is sustained, the number of youth with diabetes will more than quadruple by 2050^33,34^. Disease progression is rapid in young people and less responsive to standard treatments^35,36^, increasing the urgency for large-scale preventative intervention. In American Samoa, over half of the population are under the age of 20^37^ and children and adolescents are at significant risk of developing diabetes. Approximately 20% of adolescents in our target age range already have at least three risk factors for cardiometabolic disease (high blood pressure, large waist circumference, high triglycerides, low HDL-cholesterol, and/or high fasting serum glucose)^38^; the high prevalence of overweight/obesity in this age group (60.1%)^39^ makes them particularly vulnerable to diabetes later in life. Our approach has the potential to address management and prevention simultaneously, a significant step toward understanding how to make population level changes in diabetes outcomes that are necessary to avoid the catastrophic burden of this condition on individuals and society. If a moderate impact on adult outcomes (in line with other intervention approaches) can be replicated or improved upon, and an impact on adolescent prevention observed, these two successes would sum to the type of population level impact that is so needed in this high risk setting.

## Prior Experience (if applicable)

**This study builds on two of our previously efficacious interventions**: the Diabetes Care in American Samoa (DCAS) study, whose *materials* will be adapted, and the Strong Together Raising Our Next Generation (STRONG) study, whose *approach to intervention delivery* will be replicated.

**Diabetes Care in American Samoa (DCAS)**: Between 2007 and 2011 Dr. Rosen (study Co-I; Miriam Hospital, Providence, RI) and colleagues partnered with the AS DOH and the Tafuna Family Health Center in American Samoa to develop and test a nurse-community health worker-led (CHW) intervention for type 2 diabetes^40-43^. The intervention was a cultural adaptation of Project Sugar 2, a successful evidence-based nurse-CHW team intervention that supported diabetes self-management in a primary care setting among African Americans in Baltimore, Maryland^44,45^. Intervention content – refined through a series of community consultations^40^, and delivered through a combination of group and one-on-one home visits to participants - was guided by patient risk and self-selected goals using a menu of **eight key educational topics**: **healthy eating, becoming active, using medication, monitoring (understanding and using information from blood glucose and blood pressure measurements), tracking progress, reducing risk (preventing complications, smoking, alcohol, foot care), healthy coping (managing stress and depression), and problem solving**^40,41^. Intervention materials (flip charts) were created based on the National Diabetes Education program and used a Precede-Proceed approach^40^, with each topic covering predisposing, enabling, and reinforcing factors.

DCAS enrolled 268 adults with type 2 diabetes and randomized participants to either the nurse-CHW intervention or a wait-list control. Depending on participant risk (based on HbA1c, blood pressure, smoking and alcohol use, and depression) a primary care-based team, comprising a nurse and four community health workers, met with participants weekly, monthly, or every three months (high, moderate, and low-risk, respectively)^41^. After 12 months of intervention (74% of participants received the expected number of visits), **HbA1c was significantly lower among intervention participants, compared with control** (b = -0.53; SE = 0.21; p = 0.03) and **intervention participants had twice the odds of making a clinically significant improvement in HbA1c** (0.5% reduction), compared to control participants (p = 0.05)^41^. While the intervention did not significantly impact blood pressure, weight, or waist circumference at 12-months, intervention recipients did report significantly greater medication adherence and primary care utilization, factors that were primarily responsible for reductions in HbA1c^41,42^ However, in the year after treatment was removed, intervention participants’ HbA1c increased by 0.88 units^46^ highlighting the need for programs that can be sustained.

Summary: The successful adaptation and implementation of DCAS demonstrates: (a) the willingness of participants in AS to engage in diabetes care interventions, (b) our ability to successfully adapt existing curricula for use in the American Samoan setting, (c) the ability of CHWs/lay educators to deliver intervention content; and (d) the potential for behavior change and improvements in glycemic control among patients with diabetes. Our proposed study is designed to improve on DCAS’ success by adapting the existing curriculum/materials (which are in the Samoan language) for delivery to adolescents and will address the major shortcoming - lack of sustainability - by equipping a family member, consistently resident in the home, with the skills necessary to continuously support the individual with diabetes. While there may be concern that delivering intervention content to a third party may ‘dilute’ intervention effects compared to DCAS, we argue the opposite because of the potential for intervention content to be delivered repeatedly in the home environment and for practical as well as emotional support to be given.

**Strong Together Raising Our Next Generation (STRONG):** Drs. Hawley and Rosen recently completed an NIH-funded [R21NR016352; PI Hawley] randomized controlled trial of a group-based intervention to reduce gestational weight gain and incidence of gestational diabetes (GDM), increase uptake of gestational diabetes screening, and prolong exclusive breastfeeding among pregnant women in American Samoa. Pregnant women with overweight/obesity who attended the prenatal care clinic at LBJTMC or the TFHC were enrolled into the study before 14 weeks gestation. Intervention recipients attended ten group prenatal care appointments (with 8-10 other women of similar gestational age) during their pregnancy. Intervention content, based on an existing group prenatal care curriculum^70^, was delivered by a midwife and two lay educators using facilitated discussion and interactive group activities. The facilitated discussion approach is based on models of cooperative learning^47,48^ and allows participants to provide and receive peer-support while gaining knowledge and skills related to explicit learning objectives^49^. The principles of this approach are grounded in constructivism and social interdependence, which motivates groups to achieve common goals^50^. The intervention proved extremely effective; gestational weight gain was 4.2kg less (9.9 vs. 14.4 kg), the incidence of GDM was 39% lower (26% vs. 43% developed GDM), and blood pressure was lower throughout pregnancy among intervention participants compared to those in usual care [manuscript under review]. Qualitative evaluations revealed that the intervention was acceptable specifically because of the group education approach; participants appreciated the exchange of ideas, social support, and the interactive nature of the education and reported feeling greater self-efficacy for behavior change because of the support of their peers [under review]. Participants and stakeholders felt that the group education approach was uniquely well suited to American Samoan culture.

Summary: The positive results from STRONG were directly attributed, by participants, clinical staff, and stakeholders, to the group model for intervention delivery, which drew on several cultural strengths. The success of this project (a) speaks to the power of the group intervention model, (b) echoes the DCAS finding that lay educators are able to successfully deliver health education, and (c) demonstrates potential to change cardiovascular risk factors (weight, blood pressure). The proposed study will build on these successes by using group-based, facilitated discussion to deliver the adolescent intervention.

# Rationale/Significance

## Rationale and Study Significance

**The number of individuals diagnosed with Type 2 Diabetes in the United States (US) has more than doubled since 2000 to over 30 million, with an additional 84.1 million living with prediabetes**^1^. Although several preventative interventions have demonstrated efficacy, they are not adequately reaching the ethnic minority groups on whom a disproportionate burden of diabetes is falling^5^.

One minority group at particular risk is Pacific Islanders (PIs). **Estimates of diabetes prevalence among PIs in the US range from 13.4 to more than 45%, compared to 9.4% in the general population^5^. They are also at greater risk of end-stage renal disease and myocardial infarction as a result of uncontrolled diabetes^51,52^**. Like many other minority groups, PIs face significant barriers to healthcare: they have lower income, are less likely to have insurance than the general US population, have poorer health literacy, and often face linguistic barriers and a lack of culturally appropriate care^5, 53-57^. Despite being among the fastest growing US population groups^8-10^, PIs are underrepresented in health research and innovations in diabetes care have been slow to reach them. Novel treatment and prevention strategies are critically needed for this group.

If the intervention is successful it has the potential to benefit individual participants by improving their diabetes knowledge, their self-management skills, risk factors, and clinical diabetes outcomes. Proof of intervention efficacy would produce a scalable program with high potential for replication in other similar, low-resource, family-centered, ethnic minority groups across the US who are the ideal beneficiaries of innovations to reduce chronic disease risk and eliminate health disparities.

## Purpose of Study/Potential Impact

The number of individuals diagnosed with Type 2 Diabetes in the United States (US) has more than doubled since 2000 to over 30 million, with an additional 84.1 million living with prediabetes. Although several preventative interventions have demonstrated efficacy, they are not adequately reaching the ethnic minority groups on whom a disproportionate burden of diabetes is falling. One minority group at particular risk is Pacific Islanders, who are underrepresented in health research and who innovations in diabetes care have been slow to reach. This randomized controlled trial will investigate whether adolescents can be engaged as agents of change within their family to improve the diabetes-related health outcomes of their family members (parents, legal guardians, or grandparents). We will test the intervention in American Samoa, where diabetes prevalence is among the worst in the US. Successful completion of our aims and proof of efficacy would produce a scalable program with high potential for replication in other similar, low-resource, family-centered, ethnic minority groups across the US who are the ideal beneficiaries of innovations to reduce chronic disease risk and eliminate health disparities.

## Potential Risks and Benefits

### Potential Risks

The risks of this investigation are considered to be minimal. The present study is asking a healthy, community sample of adolescents randomized to the intervention to transmit knowledge and to provide support to a family member living with type 2 diabetes. The intervention will equip adolescents with knowledge about diabetes, coping strategies specific to the condition, information about the importance of medication adherence, and healthful diet and physical activity practices among other topics – all of which we hope will be transmitted to the adult participant paired with them for the study. We will have no contact with the adult participants during the intervention aside from for research visits and they will continue with their usual diabetes care, under the supervision of a licensed health practitioner.

The primary risk of participation is loss of confidentiality; adolescents will attend intervention sessions in groups, while adults will be asked to participate in a focus group at the completion of the intervention period. Given that the intervention and control educational sessions will happen in groups, there may be some discomfort associated with discussing personal circumstances or experiences in the presence of other adolescents, especially since we will randomize to groups based on school attendance (given the close knit social structure in American Samoa, it is highly likely that there will be others in the group who they know). The consent process will clearly explain the presence of others in the group sessions and adolescents/adults will be given the opportunity to express any concerns about this at that time. If an adolescent realizes later that they are not comfortable in a group setting, they will be free to withdraw. There are inherent concerns about confidentiality in a group setting, but we believe that the potential benefits of this design outweigh the risks. Intervention facilitators will be trained to address these issues with participants and explain the need for trust and confidentiality among groups. The physical measures that will be collected (namely blood pressure and HbA1c) may present additional risks to patients (temporary discomfort/bruising).

### Potential Benefits

There are a number of potential benefits of this research. If the intervention in successful, participants randomized to the intervention may experience direct health benefits: improved glycemic control, lower blood pressure, modest weight loss, healthful changes to diet, physical activity, and sleep, reductions in stress, and improved medication adherence. Any or all of these factors may have slow diabetes progression (adults) or prevent the onset of diabetes (adolescents). The completion of study assessments/questionnaire measures alone may provide a point of reflection for all study participants. Further, all participants may feel a sense of satisfaction that they are participating in a research study that can help identify strategies for diabetes prevention/management among fellow American Samoans and others more broadly. Information gleaned from this investigation could have important implications for future innovations in diabetes intervention. These benefits are believed to outweigh the potential risks associated with participating in this study.

# Study Purpose and Objectives

## Hypotheses

1. We hypothesize that adolescents will serve as effective conduits of knowledge/agents of change for their paired family member. Therefore, we anticipate observing improvements in **family members’** diabetes outcomes: glycemic control (HbA1c), BMI, and waist circumference.
2. While the focus of the intervention will be solely on improving their family member’s diabetes outcomes, we hypothesize that exposure to the intervention will also result in **positive health behavior change among adolescents** themselves (improved HbA1c, BMI, and waist circumference).

## Primary Objective

The primary objective of this study is to examine the efficacy of an adolescent-focused diabetes intervention (delivered solely to adolescents) in improving the diabetes outcomes of a paired family member (parent/legal guardian, grandparent) with diabetes. Outcomes will include the **family members’** glycemic control (HbA1c), body mass index (BMI) and waist circumference.

## Secondary Objective (if applicable)

The secondary objectives of the study are as follows:

1. To assess the preliminary efficacy of the program in reducing **adolescent** risk factors for diabetes (HbA1c, BMI, blood pressure, waist circumference)
2. To evaluate implementation outcomes (acceptability, feasibility, reach, fidelity) and factors influencing sustainability (program costs, likelihood of adoption)

# Study Design

### General Design Description

We will recruit and randomize n=160 dyads (an adolescent (without diabetes) and a parent or grandparent diagnosed with diabetes). **Dyads will be randomized 1:1 to the intervention or a control condition**. Adolescents from dyads randomized to the intervention group will participate in 12 group-based intervention sessions delivered over a period of six months. Adolescents randomized to the control group will be matched for contact and receive a non-diabetes focused leadership and life skills curriculum over the same six-month period. **Aside from planned research assessments we will have no contact with the adults in the dyad, who will proceed with their usual diabetes care.** To test our hypothesis that adolescents receiving the intervention will be effective conduits of diabetes knowledge and will support their paired adult in the adoption of self-care strategies, our primary efficacy outcomes will be adult glycemic control and cardiovascular risk factors (BMI, blood pressure, waist circumference). Secondarily, since we believe that exposure to the intervention may encourage positive behavior change in the adolescent themselves, we will measure adolescent glycemic control, cardiovascular risk factors, and risk behaviors**. Outcomes will be measured at baseline, after the active intervention phase (six months post-randomization) and at 12-months post-randomization, to examine maintenance of intervention effects in the absence of contact.** We will collect implementation outcomes to examine feasibility, acceptability, cost and sustainability**.**

### Study Date Range and Duration

The study will take place over a period of **three years** (October 2021 to September 2024). Recruitment is anticipated to begin in February 2022 and the final study assessments will take place in March 2024. The remaining study period will be used for analyses and dissemination of study findings.

### Number of Study Sites

There will be **four study sites**:

1. Yale University (School of Public Health, School of Medicine)
2. American Samoa Community College (where active data collection will be based)
3. University of Pittsburgh (analysis of de-identified data)
4. The Miriam Hospital, Rhode Island (analysis of qualitative evaluation data. The PI at the Miriam Hospital, Dr. Rosen, will have contact with participants during interviews completed on site in American Samoa)

## Outcome Variables

### Primary Outcome Variables

Primary outcome variables will be measured among **family members** (parents, legal guardians, grandparents) of the adolescents taking part in the intervention. Outcomes will be:

1. Glycated hemoglobin (HbA1c); a measure of glycemic control over an ~3 month period. HbA1c is expected to improve if the intervention is efficacious since the intervention targets diabetes education, medication adherence, and lifestyle change.
2. Body Mass Index (BMI); a measure of weight proportional to height. BMI is expected to improve if the intervention is efficacious because of the focus of the intervention on lifestyle change (diet and physical activity).
3. Waist circumference; a measure of central body fatness. Waist circumference is expected to improve if the intervention is efficacious because of the focus of the intervention on lifestyle change (diet and physical activity).

### Secondary and Exploratory Outcome Variables (if applicable)

Secondary outcome variables will be measured among the **adolescents** receiving the intervention. Outcomes will be:

1. Glycated hemoglobin (HbA1c); a measure of glycemic control over an ~3 month period. HbA1c is expected to improve if the intervention is efficacious since the intervention targets diabetes education, medication adherence, and lifestyle change.
2. Body Mass Index (BMI); a measure of weight proportional to height. BMI is expected to improve if the intervention is efficacious because of the focus of the intervention on lifestyle change (diet and physical activity).
3. Waist circumference; a measure of central body fatness. Waist circumference is expected to improve if the intervention is efficacious because of the focus of the intervention on lifestyle change (diet and physical activity).

We will collect a number of process measures to determine how the intervention may impact these outcomes. These will include: social/emotional relationship between the adolescent and family member taking part, self-care behaviors among both adolescent and adult (diet, physical activity, stress, sleep, smoking, alcohol use), diabetes literacy among adolescents and adults, and medication adherence and healthcare utilization among family members only. Process measures will be collected with both quantitative and qualitative approaches.

We will also collect information about intervention acceptability, feasibility, reach, fidelity, and factors influencing sustainability. These data will be collected from intervention participants as well as local stakeholders using quantitative and qualitative approaches.

## Study Population

The study will enroll n=160 dyads (n=320 total individuals). Each dyad will comprise an adolescent (14-17 years; healthy, without diabetes) and a paired family member (parent, legal guardian, grandparents with diabetes). Recruitment efforts will target adolescents and will be focused on four Department of Education-run high schools.

We will also enroll up to n=25 stakeholders – individuals who are knowledgeable about either the intervention and its delivery and can provide evaluation, or those who are knowledgeable about the American Samoa health system, diabetes care, or community health in general.

### Number of Participants

The study will enroll **n=160 dyads (n=320 total individuals).** Reflecting the exploratory nature of the study, the sample size was determined based on both feasibility and statistical power. In an intent-to-treat analysis we will have approximately 40% power to detect a standardized between-group difference of 0.3 (i.e. the mean outcome differs between intervention and control by 0.3 standard deviations or approximately 0.68%) and >80% power to detect a standardized between-group difference of 0.5 (approximately 1.13%). We will have >90% power to detect changes in categorical outcomes (% participants meeting recommended dietary intake, for example).

Up to n=25 stakeholders will complete semi-structured interviews as part of the evaluation process.

### Eligibility Criteria/Vulnerable Populations

Eligibility will be determined by trained study staff with the assistance of Dr. Rabin if a clinical opinion is required to determine eligibility. Adolescents (14-17 years), who are considered a vulnerable population will be enrolled, along with a paired family member (parent, legal guardian, grandparent) since they (adolescents) are the primary targets of the proposed intervention. A parent or legal guardian will be required to provide informed consent for the adolescent to participate and the adolescent will have to give their own informed consent.

**Inclusion and exclusion criteria for trial participants** are as follows:

**Inclusion Criteria:**

- Adolescent (14-17 years) and parent/legal guardian/grandparent (no age restriction) who share the same household
- Both participants willing and able to consent to participation
- Parent or guardian willing to consent to adolescent’s participation
- Samoan ethnicity
- The adult must have received a diagnosis of T2DM by a clinician at least 12 months prior to enrollment (to avoid any immediate post-diagnosis behavior change masking effects of the intervention)
- Adult HbA1c ≥ 6.5% (indicating a need for intervention to support glycemic control)
- The adult must have been prescribed medication (either tablets or insulin) whether they are currently taking the medication or not.
- Adolescents must be willing/able to participate in group sessions after school or on Saturday mornings

**Exclusion Criteria:**

- Adult women/adolescent girls will be ineligible if they are planning to become pregnant during the study period (and will be excluded from analysis if they become pregnant while enrolled).
- Both participants (adolescent and paired adult) will be deemed ineligible if either of the pair are planning to leave American Samoa in the next 18 months
- Participants will be excluded if they report any of the following:
  - Uncontrolled hypertension (systolic >180 mmHg or diastolic >105 mmHg)
  - Heart attack, stroke, or transient ischemic attack in the past year
  - Treatment for cancer
  - Chest pain or shortness of breath with minimal activity
  - Chronic lung disease, or asthma requiring home oxygen therapy
  - Contraindications to moderate physical activity
  - Inability to read/speak Samoan and/or English.
- Adolescents will be excluded if they have overt diabetes (HbA1c ≥6.5%) based on point-of-care testing during the screening process, but will remain eligible if they appear to have prediabetes (HbA1c >5.7%), since the intervention is likely to benefit them, particularly in the absence of any existing programs in American Samoa to support lifestyle intervention in adolescence.

Any participant determined to have uncontrolled hypertension or a new diagnosis of diabetes during screening will be referred to the health system for follow up.

**Inclusion criteria for stakeholders** will include:

- Age >18 years
- Willingness and ability to consent to participation
- Familiarity with either the intervention and it’s delivery or the American Samoa health care system, diabetes care, or community health in general.
- Ability to speak English

1. **Study Methods/Procedures**

## Study Procedures

### Data Collection

**Recruitment.** We will recruit and randomize n=160 dyads. Each dyad will include an adolescent without diabetes and their parent or grandparent, who is currently receiving treatment for diabetes. Since adolescents will be the recipients of the intervention, they will be the target of recruitment efforts. We will partner with four Department of Education-run high schools (Leone High School [enrollment 675 students], Nu’uli High School [265 students], Tafuna High School [1202 students], Samoana High School [946 students]) and advertise the study to adolescents through assemblies, visits to classrooms in the age-range of interest, and posters placed around the schools. Interested participants will contact study staff by phone, or deposit an expression of interest in designated boxes at the school. Adolescents will be encouraged to discuss their participation with the member of their family who they wish to participate with before contacting study staff.

**Screening and Eligibility**. Project staff will visit interested adolescents and their family members in their home to explain the study, determine eligibility, and gain informed consent. Questionnaires, blood pressure, and HbA1c screening (using a point-of-care A1cNow device, Bayer Healthcare) will be used to determine eligibility. See section 5.3.1 for inclusion and exclusion criteria.

**Consent & Randomization.** If both members of the adolescent-adult pair are eligible based on the criteria described above and HbA1c screening, they will consent separately to participation (but both must agree). Adults will complete written informed consent forms; adolescents will give their written assent and will also be required to provide parental/legal guardian consent. Baseline research assessments will be completed either the same day or at another time convenient to the pair, but before randomization. **Randomization to the intervention or control condition will be school-based (n=2 schools in each condition), to reduce the likelihood of contamination between groups, and stratified by self-reported gender to recruit equal numbers of male and female participants.** Participants will be given a schedule and instructions for attendance (including dates/times of sessions) based on their group assignment.

**Intervention and Control Group Activities.** All session content will be delivered by our Project Director at the American Samoa Community College, a central location to the four high schools (ASCC). We will place adolescents into a group with nine others (n=10 total; gender-balanced). Individuals will attend all sessions with the same group. Groups will meet once every two weeks (after school/on Saturday mornings) for six months (12 sessions total), with sessions lasting approximately 90 minutes each. A research assistant (RA) will support Project Director in delivering the group intervention sessions and will work with them to establish a process for making reminder calls/texts to encourage and track attendance. To promote sustainability and increase the likelihood of adoption and scale up at the end of the grant period, transport will not be provided to the adolescents, since public transport (which most adolescents use to attend school) is regularly and very cheaply available to this age group (two schools are within walking distance of ASCC). For the same reason, we will not incentivize participation in the group sessions but will provide healthy snacks (which we have used effectively in other interventions to model portion size); both groups will receive the same snacks, but only the intervention group will discuss them as an example of portion size.

The curriculum for the intervention group is described in the Table below. Adolescents randomized to the control group will meet for the same number of sessions, but will receive only the leadership and life skills components of the intervention. Their sessions, which will last an hour, will be focused on building capacity for leadership and applying it to considering their future life and career goals and motivations. Facilitated discussion and experiential learning activities will be applied in the same way, but the focus will not be on health generally, or diabetes explicitly.

**Outcome Assessments.** Participants (adolescent and adult) will complete assessments prior to randomization (baseline), after the active intervention phase (6-months post-randomization) and after a six-month, non-active maintenance phase (12-months post-randomization). This approach will allow us to observe both intervention and longer-term maintenance effects on physiological and behavioral outcomes. All assessments will be conducted by a trained research assistant **blinded to group assignment**. Assessments will take place in participants’ homes and each participant will receive $20 for completing the activities at each time point.

**Table 1: Overview of the Proposed Intervention Curriculum**

| **Session** | **Educational Focus**  ***Barriers to be addressed*** | **Leadership Activity** | **Experiential Learning Opportunity** |
| --- | --- | --- | --- |
| 1 | What to expect; what is diabetes?  T2DM risks, pathophysiology, symptoms  *Barrier: diabetes/general health literacy* | Who do you admire and why? | Using data: how big is the problem? |
| 2 | How do you treat diabetes?  *Barrier: Medication Adherence* | Planning strategies: breaking down tasks |  |
| 3 | Keeping track, identifying and responding to emergency situations  *Barrier: medication adherence, self-monitoring, health numeracy* | The guessing game: using open and closed questions | Developing new tools for medication tracking & self-monitoring |
| 4 | Getting the right healthcare (primary care utilization)  *Barrier: engagement in care, cost (low cost/free options will be identified)* | How do leaders lead? | Hospital visit/Group Discussion with healthcare providers  *Barrier: culturally competent care (participants will learn how to communicate with providers and to advocate for patient care; providers will hear adolescent feedback about current barriers to care)* |
| 5 | Making the best health decisions (health behavior theory)  *Barrier: lack of prioritization of health* | “I care” values: reflecting on why we care | Self-assessment exercise: how do you make everyday decisions? |
| 6 | All about food (dietary change, mindful eating)  *Barrier: cost, health literacy* | Brainstorming for change: solving complex problems | Cooking demonstrations |
| 7 | Reaching activity goals  *Barrier: motivation, health literacy* | Ten keys to effective listening | ‘Training-the-trainers’ group exercise session |
| 8 | How do we measure health?  *Barrier: health literacy and numeracy* | Emotion awareness: identifying complex emotions | Measuring blood pressure/glucose |
| 9 | The role of sleep and stress  *Barrier: health knowledge* | A quick quiz: how to evaluate your experiences? | Keep a sleep diary |
| 10 | Breaking down barriers (Problem solving)  *Barrier: cost, culturally competent care* | Why and why not? Effective reasoning skills |  |
| 11 | Making sure medicines work (medication adherence)  *Barrier: medication adherence, diabetes numeracy* | Practicing speaking and listening in families | Evaluating tools developed in session 3: did they work? |
| 12 | Maintaining healthy habits  *Barrier: motivation, sustainability* | Future thinking: how to set your course |  |

^a^Led by local design start-up company; ^b^Led by South Pacific Watersports (safe exercise for all ages)

**Primary Outcomes.** The primary outcomes are **adult glycemic control (HbA1c) and cardiovascular risk factors (BMI, waist circumference, blood pressure).** *Physical measures*: We will measure HbA1c using a point-of-care device (A1cNow, Bayer). The measure collected during the recruitment and screening process (to determine eligibility) will be used as the baseline measure. Body weight and height will be measured using a SECA portable stadiometer and Tanita HD 351 digital scale, respectively, and will be used to calculate BMI. Waist circumference, a proxy for visceral adiposity, will be measured using a cloth measuring tape. Blood pressure will be measured in triplicate on the non-dominant arm, using an automated sphygmomanometer (Omron HEM 907XL) at five-minute intervals.

**Secondary Outcomes.** The secondary outcomes are **adolescent risk factors for diabetes (HbA1c, BMI, blood pressure, waist circumference).** The same procedures described above will be used to measure key outcomes in adolescents.

**Covariates and Process Measures.** Since it is clear that social and environmental factors have a considerable impact on the potential effectiveness of health interventions, we will collect data from participating families to assist in determining why the program is or is not effective (covariates) and by what behavioral pathways the intervention may have exerted its effect (process). Because the intervention approach uses adolescents as agents of change, we will also attempt to measure communication between the adolescent-adult pair, and what information the adult recalls receiving from the adolescent (process). Covariates collected at the *baseline assessment only* will include number of individuals sharing the household [as a measure of household resources], household socio-economic position (using self-reported income and a household assets inventory, and food security. We will also record participant age, self-reported gender, and biological relationship between the pair (parent and child/grandparent and child) since these characteristics may influence outcomes. We will measure the social/emotional relationship among the pair at each assessment time point (so that we may examine change with participation) using the Network of Relationships Inventory. Process measures will include self-care behaviors (diet, physical activity, stress and sleep, smoking and alcohol use, and diabetes literacy (all measured in both adult and adolescent) and medication adherence and primary care utilization (adults only). Dietary intake will be measured using the National Health and Examination Survey (NHANES) dietary screener, which documents intake of fiber/whole grains, added sugars, dairy products, red meat, fruits and vegetables, and fast-food (which will be the primary targets of the nutritional education provided during the intervention). Physical activity will be estimated using the WHO Global Physical Activity Questionnaire (GPAQ) which we have translated and used extensively in the Samoan setting. The PHQ-9 (used in DCAS) and Cohen’s Perceived Stress Scale will be used to measure depressive symptoms and stress, respectively, while the MESA Sleep questionnaire will capture sleep duration and perceived quality. Participants will self-report smoking and alcohol consumption. Diabetes numeracy, literacy and knowledge will be assessed using the Diabetes Numeracy Test (DNT), Literacy Assessment for Diabetes (LAD), and the Diabetes Knowledge Scale^.^ Among adolescents only, we will also measure perceived risk of developing diabetes using an adapted version of the Risk Perception Survey for Developing Diabetes^.^ Among adults only, medication adherence will be measured with a modified version of the Hill-Bone High Blood Pressure Therapy Scale that was adapted for this setting and for diabetes during an earlier study. The Diabetes Distress Scale will be also be used to document any perceived change in control over their condition. Medical records will be reviewed to determine health care utilization of the adult participants (primary care, emergency care, inpatient stays, and preventative medicine [dental, foot clinic, mental health services]) and medication adherence (prescriptions and refills). All assessments proposed here have been previously completed in this setting and the questionnaire measures are available in Samoan.

We will develop additional, new process tools specifically for this study. As time spent by the adolescent providing material and emotional support to the adult may be difficult to quantify, we will ask adults to rate these dimensions of support on a scale of 1-10 at each time point. We will describe emotional support as including factors such as empathy, understanding, and responsiveness; instrumental support will be described as tangible helping (for example preparing medication, providing reminders, accompanying them during activities, cooking together, etc.). After the active intervention is complete (6-months post-randomization) we will ask adults assigned to the intervention arm to complete a questionnaire that lists the topics covered in the adolescent group sessions; participants will report whether the adolescent discussed these topics with them at any time during the past six months. We will also conduct semi-structured interviews with 20 pairs of participants – specifically, the 10 with the greatest and the least observed change in adult HbA1c. Interviewing the pair together, we will focus on what aspects of their communication and behavior changed over the course of the intervention and their beliefs about why they had more or less success. Similar interviews will be conducted with the control group (n=20 pairs) to determine any potential impact of participating in the control condition.

**Intervention Evaluation: acceptability, feasibility, reach, fidelity, and factors influencing sustainability (program costs, likelihood of adoption).** We will integrate evaluation activities throughout the implementation of the intervention to capture outcomes critical to understanding the potential for adoption into practice and future scaling in American Samoa and more broadly. Using best practices from implementation science^108,109^ we will document acceptability, reach and adoption, feasibility, fidelity, cost, and potential for sustainability and scale-up. Activities to be used and the timing of data collection are described in **Table 2** below.

| **Outcome** | **Evaluation Measures** | **Participants/Timeline** |
| --- | --- | --- |
| **Acceptability** | Acceptability will be determined based on *participant satisfaction* which we will measure using:   - Structured questionnaires: perceived participation burden, opportunity costs, experience of participation, attitude toward intervention and associated activities - Focus Group Discussions (FGDs): FGDs will be conducted with adolescent (n=4 groups) and adult participants separately (n=4 groups). Topics: similar to the questionnaire measures - Stakeholder interviews: (n=10-15; research staff responsible for intervention delivery, health system leadership) experiences of intervention implementation, burden of delivery, feedback from participants - Brief interviews with participants who withdraw, primary reasons for discontinuation | Intervention participants and stakeholders; after completion of the active intervention phase (6m post-randomization)  Study non-completers (upon notification of withdrawal) |
| **Reach and Adoption** | Measured using elements of the RE-AIM framework reach and adoption will be estimated using:   - Recruitment metrics: number of potential participants included/excluded, % who participate, characteristics of participants vs. general population - FGDs and stakeholder interviews: study staff experiences of recruitment, stakeholder perceptions of potential adoption among community organizations | Stakeholders; upon completion of recruitment activities and after implementation is complete |
| **Feasibility** | Feasibility will be determined based on *recruitment, enrollment,* and *retention* rates as well as *adherence* and *engagement* with intervention activities   - Retention and adherence metrics and drop-out interviews: % of participants who do not complete the study; number of sessions attended - Brief interviews with participants who withdraw, barriers to participation - Completion of measurement tools: % of missing data - Research staff interviews: challenges with program delivery or measurement of outcomes | Study non-completers (upon notification of withdrawal); Health educators (after implementation is complete) |
| **Fidelity** | Fidelity to the intervention curriculum (for both intervention and control groups) measured using:   - Observations/audio recordings of sessions: adherence to planned content, completion of planned experiential learning activities, quality of delivery (interventionist enthusiasm, communication style, and confidence) - Fidelity checklists: intervention dose (duration of study sessions, interaction with participants outside of formal sessions) - Structured questionnaires: adolescent report of achieving learning outcomes (short (2-3 question) surveys will be completed after each group session) | Study PI (review of session content)  Interventionists (after each session)  Adolescents (after each session) |
| **Cost** | Program costs will be evaluated using a *micro-costing approach* to generate estimated per-participant costs:   - Structured questionnaires: participants (adolescent and adult) will estimate time and resources spent participating (travel, food costs, etc.), medical costs incurred - Medical records: participant medical costs - Stakeholder interviews/questionnaires: health system accounting departments will provide estimates of direct medical costs and non-medical costs, personnel costs (salaries), intervention materials, facility-level overhead costs | Intervention Participants; after the active intervention phase  Stakeholders; after implementation is complete |
| **Sustainability and Potential to Scale** | Assessment of potential for scale-up and sustainability will be guided by WHO steps for developing a scaling-up strategy (steps 3 & 4) and will focus on *environment* and *human capacity*.   - Stakeholder interviews: identification of key stakeholders for scale-up, political/policy connections needed, related initiatives that could be leveraged, likely barriers to scaling, leadership and advocacy potential within the health system, experience with successful scaling of other programs, stability of human resources, motivation of health system leadership to sustain the program | Stakeholders; after implementation is complete |

**Measures Included for Committee Review:** We have included questionnaires to be used during research assessments for initial committee review. Questionnaires specific to the evaluation and guides for focus group/semi-structured interviews are being developed by our community advisory board and will be informed by the initial implementation of the intervention. These materials will be submitted to the Yale and American Samoa IRBs via a protocol modification before use.

### Adverse Events Definition and Reporting

Adverse event (AE) monitoring will occur at all assessment time points and spontaneously by participant report.

The following potential AE (and associated definitions) have been identified. Causality of adverse events will be determined according to the best judgment of the PI and study physicians Dr. Tracy Rabin, using all available information to determine if the event is related to the study.

High Blood Pressure

If any participant’s measured blood pressure is ≥ 160/110, study staff will call a hospital-based clinician for a referral immediately and have a plan for follow up in place before the participant is allowed to leave the research assessment.

If measured blood pressure is ≥ 140/90 (but less than 160/100), study staff will provide a referral note and instruct participants to make an appointment with a primary care clinician to arrange follow up. Additionally, study staff will notify American Samoa-based clinicians by phone after the study visit.

If blood pressure meets the above criteria on first reading a second measurement will be taken. If one measurement is above and one below a third measurement will be taken. At least two blood pressure readings must meet criteria for an AE alert.

Marked Decreases in HbA1c [Adults Only]

Some studies suggest that if a patient has poorly controlled diabetes (HbA1c >9%) for many years and then rapidly controls their diabetes (a decrease of >3%), it may hasten the onset of retinopathy or cardiac morbidities.

If HbA1c has decreases by 2.0% or more during the six-month intervention period, participants will be referred to primary care clinicians for assessment and follow up. Ther participants diabetes care provider will be notified immediately and will work with primary care staff to ensure appropriate follow up.

Serious Adverse Events (SAE), are defined as an untoward medical occurrence, whether associated with study participation or not, that results in one of the following:

1. Death
2. Life-Threatening Event
   - Life-threatening events are defined as those that in the view of the research staff and PI put the individual participant at imminent substantial risk of dying
3. Hospitalization (initial or prolonged)
4. Disability or Permanent Damage
   - If the adverse event resulted in a substantial disruption of the participants ability to conduct normal life functions.

**For each of the AEs described above, the following scale will be used by the PI and study clinician to identify study relatedness:**

0 = Unrelated

1= Unlikely

2= Possibly related

3= Probably Related

4=Definitely Related

Only AEs that are serious, unanticipated, and at least possibly related to participation in the study will be reported to the IRBs at Yale University and the American Samoa Department of Health per their reporting requirements.

## Study Schedule

Adolescents enrolled in the study will have 15-16 interactions with the study as follows: the screening/consent visit (60-90 minutes, which may be divided into two visits depending on participant preference), research assessments at 6- and 12-months post-randomization (60 minutes each), and 12 intervention sessions (90 minutes each). Paired family members will have three interactions with the study – they will participate in the screening/consent visit (60-90 minutes, with the adolescent) and research assessments at 6- and 12-months post-randomization (60 minutes each).

Stakeholders who are invited to take part in the evaluation process will complete one semi-structured interview with research staff (Dr. Hawley or Rosen). This interview will take between 45 and 75 minutes to complete.

## Informed Consent

*Intervention participants*:

If both members of the adolescent-adult pair are eligible based on the criteria described above and HbA1c screening, they will consent separately to participation (but both must agree). Adults will complete written informed consent forms; adolescents will give their written assent and will also be required to provide parental/legal guardian consent given their age. Consent forms will be provided in both English and Samoan (side-by-side as has been participant preference in our other studies). Participants will be free to withdraw at any time during the study period.

Adolescent and adult study participants will complete the consent process together; we will explain that we are interested in learning about whether adolescents can be equipped to support their family members in dealing with their diabetes. A general overview of what it entails to be in a research study and how randomization works will be reviewed with potential participants before a description of this specific study is provided. Specifically, we will explain to participants what this study would entail, explain that the adolescent would be the one to actually receive educational sessions, but that adults will continue with their usual care, and that we would be visiting their home three times over the course of the intervention to conduct research assessments. They will be informed that they will be enrolled in the study for a year, and will also be asked to provide feedback on the process during focus groups and/or semi-structured interviews. A description of all sources of materials to be collected at each assessment and throughout the intervention will be reviewed with the pair. Finally, they will be informed that each assessment will take about 40 minutes (each) for them to complete, and that they will be compensated $20 at each of the assessment visits (for $60 total) [this amount reflects the compensation recommended during preliminary discussions with the American Samoan IRB]. After this description of the study is completed, understanding will be checked (by the use of open ended questions and asking participants to describe in their own words what they are agreeing to), and any questions are answered, a trained member of the research team will obtain informed written consent from the participants. Adult participants will provide written consent, adolescents will provide written assent and biological parent/legal guardian consent will be sought for the adolescent’s participation.

*Stakeholders*:

Stakeholders will be asked to provide written consent for their participation in semi-structured interviews. Research staff will explain the objectives of the interview, provide an overview of interview questions, and will answer any questions participants may have about their enrollment in the study before the written consent is obtained.

The following consent materials are submitted with this protocol:

- Adolescent informed consent form
- Parental consent for adolescent participation
- Adult informed consent form
- Stakeholder consent form

### Screening (if applicable)

Project staff will visit interested adolescents and their family members in their home to explain the study, determine eligibility, and gain informed consent. Questionnaires, blood pressure, and HbA1c screening (using a point-of-care A1cNow device, Bayer Healthcare) will be used to determine eligibility. **Inclusion criteria**: Eligible participants will be an adolescent (14-17 years) and parent/legal guardian/grandparent who share the same household; both participants must be willing and consent to participating in the program together and be of Samoan ethnicity. The adult must have received a diagnosis of T2DM by a clinician at least 12 months prior to enrollment (to avoid immediate post-diagnosis behavior changes masking effects of the intervention), have an HbA1c ≥6.5%, and have been prescribed diabetes medication (either tablets or insulin) whether they are currently taking medication or not. To be included, adolescents must be willing/able to participate in group sessions after school or on Saturday mornings. There will be no additional physical/ biochemical inclusion criteria, although given the population prevalence we expect the majority of participants (>65%) to be overweight/obese and up to 30% will have prediabetes based on HbA1c^46^. **Exclusion criteria**: Adult women/adolescent girls will be ineligible if they plan to become pregnant during the study period (and will be excluded from analyses if they become pregnant while enrolled). Both participants will be deemed ineligible if either are planning to leave American Samoa in the next 18 months or if they report any of the following: uncontrolled hypertension (systolic >180 mmHg or diastolic >105 mmHg), heart attack, stroke, or transient ischemic attack in the past year, treatment for cancer, chest pain or shortness of breath with minimal activity, chronic lung disease, or asthma requiring home oxygen therapy, inability to read/speak Samoan and/or English or contraindications to moderate physical activity. Adolescents will be excluded if they have overt diabetes (HbA1c ≥6.5%) based on testing during the screening process, but will remain eligible if they have prediabetes (HbA1c >5.7%), since the intervention is likely to benefit them, particularly in the absence of any existing programs in American Samoa to support lifestyle intervention in adolescence. Any participant determined to have uncontrolled hypertension or a new diagnosis of diabetes will be referred to the health system for follow up.

### Recruitment, Enrollment and Retention (if applicable)

We will recruit and randomize n=160 dyads. Each dyad will include an adolescent without diabetes and their parent or grandparent, who is currently receiving treatment for diabetes. Since adolescents will be the recipients of the intervention, they will be the target of recruitment efforts. We will partner with four Department of Education-run high schools (Leone High School [enrollment 675 students], Nu’uli High School [265 students], Tafuna High School [1202 students], Samoana High School [946 students]) and advertise the study to adolescents through assemblies, visits to classrooms in the age-range of interest, and posters placed around the schools. Interested participants will contact study staff by phone, or deposit an expression of interest in designated boxes at the school. Adolescents will be encouraged to discuss their participation with the member of their family who they wish to participate with before contacting study staff.

Enrollment into the study will be voluntary. Adolescents and adults will participate in a screening and consent process together; we will explain that we are interested in learning about whether adolescents can be equipped to support their family members in dealing with their diabetes. A general overview of what it entails to be in a research study and how randomization works will be reviewed with potential participants before a description of this specific study is provided. Specifically, we will explain to participants what this study would entail, explain that the adolescent would be the one to actually receive educational sessions, but that adults will continue with their usual care, and that we would be visiting their home three times over the course of the intervention to conduct research assessments. They will be informed that they will be enrolled in the study for a year, and will also be asked to provide feedback on the process during focus groups and/or semi-structured interviews. A description of all sources of materials to be collected at each assessment and throughout the intervention will be reviewed with the pair. Finally, they will be informed that each assessment will take about 40 minutes (each) for them to complete, and that they will be compensated $20 at each of the assessment visits (for $60 total) [this amount reflects the compensation recommended during preliminary discussions with the American Samoan IRB]. After this description of the study is completed, understanding will be checked (by the use of open ended questions and asking participants to describe in their own words what they are agreeing to), and any questions are answered, a trained member of the research team will obtain informed written consent from the participants. Adult participants will provide written consent, adolescents will provide written assent and biological parent consent will be sought for the adolescent’s participation if the adult they are enrolling with is not a parent. The consent form will be provided in both English and Samoan. Participants will be free to withdraw at any time during the study period.

To maximize retention of participants for the duration of the proposed study we will employ a number of strategies that have been successful in our other studies in this setting: (1) we will obtain detailed primary and secondary contact information from participants and will update/confirm these contact details at each assessment visit; (2) we will schedule study visits at convenient times of the day or at weekends, (3) we will conduct assessments in the participant’s home or at a place most convenient for them; (4) we will use several means to remind participants of follow-up assessments, including appointment cards, telephone calls, text messages, and home visits if necessary; (5) we will provide regular study updates and opportunities for engagement through our dedicated Facebook page (@YaleOlaga); and (6) we will offer small incentives for participation in the research assessments ($20 for each assessment, $60 total); (7) we will employ Samoan research assistants with excellent interpersonal skills, organizational skills, and ability to demonstrate the empathy and cultural sensitivity needed for participant engagement. They will have received extensive training on approaches to retention of study participants before the study begins. Finally, (8) we will maintain a community advisory board for this study. The board is composed of adolescents in the same age range as those to be enrolled in the intervention and they will continue to advise us about the best strategies to ensure continued engagement with study protocols. We will monitor retention patterns continually using a comprehensive participant tracking system to record attempted contacts and missed and attended appointments and will review this information regularly.

If either an adult or an adolescent is permanently lost to follow up or must be excluded (for example, moves out of American Samoa, becomes pregnant, or develops a medical condition that precludes continued participation, among others) the remaining member of the pair will be allowed to continue in the intervention and complete the assessment visits as planned. If an adolescent is lost to follow up post-randomization after receiving even some of the intervention, there may be measureable effects in the adult family member (although the circumstances of adolescent loss to follow up will be reviewed before including the adult data in data analysis). If the adult participant is lost to follow up, we will still be able to address secondary outcomes (adolescent risk factor reduction).

Participants who withdraw from the study will be asked whether their existing data may be included in future analyses. If they wish for their data to be removed, it will be removed permanently from the dataset and all further analysis. Because one of the primary goals of this study is to establish intervention acceptability, we will ask participants who withdraw to share their reasons for withdrawal if they are willing. We will still routinely invite participants who withdraw to any events that communicate study findings.

Stakeholders who participate in evaluation activities will be recruited by snowball sampling. We will begin with the local research staff that will oversee implementation of the study and the diabetes clinical care coordinator in American Samoa. We will then ask each of the participants to suggest others who may fulfill the eligibility criteria. Research staff will contact potential participants by phone, email, or by in person visit to their place of work to invite them to learn more about the study. If individuals are interested in learning more, staff will schedule an appropriate time to review the consent form and study procedures before gaining informed consent for participation.

### Study Visits

**N.B.** This section describes the research activities completed by adolescent-adult dyads. In addition, adolescents will participate in 12 group sessions over the course of 6 months, where they will receive either a diabetes-focused curriculum or leadership and life skills training.

**Research Assessments:**

1. Recruitment & Screening Visit (~40 minutes)

| Research Activity | Adolescent | Adult Family Member |
| --- | --- | --- |
| Explanation of study purpose and procedures | X | X |
| HbA1c measurement | X | X |
| Blood Pressure Screening | X | X |
| Eligibility Questionnaire (health conditions, plans to leave American Samoa, contraindications to physical activity) | X | X |
| Consent form review and signature | X | X |

1. Baseline Assessment (~1 hour)

| Research Activity | Adolescent | Adult Family Member |
| --- | --- | --- |
| Physical measurements (height, weight, waist circumference) | X | X |
| HbA1c measurement | X | X |
| Blood Pressure Screening | X | X |
| Demographic Questionnaire (participant and household characteristics, socioeconomic position, food security) |  | X |
| Network of Relationships Inventory | X | X |
| Self Care Questionnaire (diet, physical activity, stress, sleep, smoking and alcohol use) | X | X |
| Diabetes Literacy Questionnaire | X | X |
| Diabetes Self-Management Questionnaire |  | X |
| Perceived Diabetes Risk Questionnaire | X |  |
| Emotional and Material Support Questionnaire |  | X |

1. 6-month Post-Randomization Assessment (end of active intervention; ~1 hour*)

| Research Activity | Adolescent | Adult Family Member |
| --- | --- | --- |
| Physical measurements (height, weight, waist circumference) | X | X |
| HbA1c measurement | X | X |
| Blood Pressure Screening | X | X |
| Network of Relationships Inventory | X | X |
| Self Care Questionnaire (diet, physical activity, stress, sleep, smoking and alcohol use) | X | X |
| Diabetes Literacy Questionnaire | X | X |
| Diabetes Self-Management Questionnaire |  | X |
| Perceived Diabetes Risk Questionnaire | X |  |
| Emotional and Material Support Questionnaire |  | X |
| Diabetes Knowledge Transfer Questionnaire (topics from the intervention discussed between the participating dyad) |  | X |
| Semi-structured Interview* | X | X |

*Interviews will be conducted with a total of 40 dyads – 20 from the intervention arm (the 10 dyads with the greatest and least observed change in adult HbA1c) and 20 from the control condition. Those participating in the semi-structured will be required to contribute an additional 45 minutes to 1 hour of their time (2 hours total).

1. 12-month Post-Randomization Assessment (end of maintenance phase; 1 hour)

| Research Activity | Adolescent | Adult Family Member |
| --- | --- | --- |
| Physical measurements (height, weight, waist circumference) | X | X |
| HbA1c measurement | X | X |
| Blood Pressure Screening | X | X |
| Network of Relationships Inventory | X | X |
| Self Care Questionnaire (diet, physical activity, stress, sleep, smoking and alcohol use) | X | X |
| Diabetes Literacy Questionnaire | X | X |
| Diabetes Self-Management Questionnaire |  | X |
| Perceived Diabetes Risk Questionnaire | X |  |
| Emotional and Material Support Questionnaire |  | X |

**Evaluation Activities:**

1. Adolescent Focus Group Discussions: Adolescent focus group discussions will take place with all groups during the final one of their 12-group sessions (~6 months post-randomization).
2. Adult Focus Groups: Adults randomized to the diabetes intervention will be asked to participate in a focus group discussion after completing their participation (12 months post-randomization). Where possible, these will be conducted in the original groups (all family members of one adolescent group will participate together).
3. Stakeholder Interviews: Stakeholders will participate in a single, semi-structured interview after the intervention delivery is complete.

## Statistical Method

### Statistical Design

Using an intent-to-treat approach, we will compare group differences in primary and secondary outcomes post-active intervention and following the maintenance phase. Generalized estimating equations (GEE) with the robust “sandwich” variance estimator accounting for the within-cluster correlation will be used to model outcomes post-active intervention and then separately following the maintenance phase. For continuous outcomes (e.g. HbA1c) we will use these models to examine differences in (1) the mean outcome and (2) the rate-of-change of the outcome between the intervention and control conditions. Each of the outcomes will be modeled individually. If significant differences between groups are identified post-intervention/maintenance, we will conduct subgroup analyses to understand the process underlying those differences using GEE. Prior to analysis, the demographic characteristics and baseline measures will be compared between the intervention and control conditions. Any measures demonstrating evidence of difference between intervention and control, in addition to any relevant participant characteristics, such as age and gender, will be included in all analyses of post-active intervention and maintenance outcomes. Since this is a pilot study to provide estimates for a larger trial, there will be no adjustment for multiple comparisons.

While this project is not adequately powered to test for these interactions (see below), we will perform exploratory analyses of gender-specific effects as we expect that intervention efficacy may vary based on (a) the adolescent’s gender, (b) the adult’s gender, and (c) the gender match between the pair. To explore this we will estimate the statistical interactions between the three gender variables defined above and intervention/control group assignment. We will compare estimated outcomes from the statistical models to see if there is, at a suggestive and qualitative level, any differences in intervention efficacy that could inform future intervention design or be further examined in larger trials.

### Sample Size Considerations

The study will enroll **n=160 dyads (n=320 total individuals).** Reflecting the exploratory nature of the study, the sample size was determined based on both feasibility and statistical power.
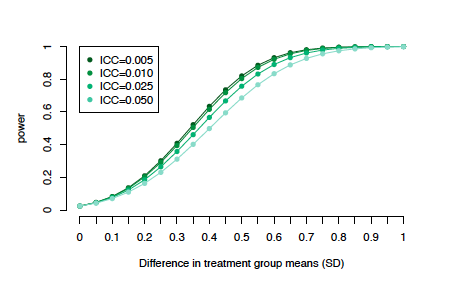
 In line with the nature of the pilot study, we recognize that we will be underpowered to detect differences in some of our physiological endpoints. We calculated estimated power curves to detect between-group differences assuming a continuous outcome GEE model with 16 groups of 10 dyads each (8 intervention and 8 control) for various effect sizes and intraclass correlations (ICC) (see **Figure**). Based on preliminary data from the DCAS study, which had an estimated ICC of 0.011, we would have approximately 40% power to detect a standardized between-group difference of 0.3 (i.e. the mean outcome differs between intervention and control by 0.3 standard deviations or approximately 0.68%) and >80% power to detect a standardized between-group difference of 0.5 (approximately 1.13%). Based on STRONG data, we will have >90% power to detect changes in categorical outcomes (% participants meeting recommended dietary intake, for example).

### Planned Analyses

Outcome Evaluation: Using an intent-to-treat approach, we will compare group differences in primary and secondary outcomes post-active intervention and following the maintenance phase. Generalized estimating equations (GEE) with the robust “sandwich” variance estimator accounting for the within-cluster correlation will be used to model outcomes post-active intervention and then separately following the maintenance phase. For continuous outcomes (e.g. HbA1c) we will use these models to examine differences in (1) the mean outcome and (2) the rate-of-change of the outcome between the intervention and control conditions. Each of the outcomes will be modeled individually. If significant differences between groups are identified post-intervention/maintenance, we will conduct subgroup analyses to understand the process underlying those differences using GEE. Prior to analysis, the demographic characteristics and baseline measures will be compared between the intervention and control conditions. Any measures demonstrating evidence of difference between intervention and control, in addition to any relevant participant characteristics, such as age and gender, will be included in all analyses of post-active intervention and maintenance outcomes. Since this is a pilot study to provide estimates for a larger trial, there will be no adjustment for multiple comparisons.

Intervention Evaluation: Quantitative data will be summarized descriptively, with paired samples t-tests and generalized linear models used for between group comparisons as appropriate. FGDs and SSIs will be transcribed and translated/back-translated as needed. Transcripts will be entered into NVivo analysis software and coded using topic areas developed from the FGD and SSI agendas and transcripts to identify broad themes and patterns related to implementation outcomes and germane to future iterations of the intervention, answering both on our *a priori* research questions (*how acceptable and feasible is this intervention? What is the potential for sustainability and scale-up?*) and themes that emerge from the data.

### Analysis of Subject Characteristics (if applicable)

Prior to any outcome analysis, the demographic characteristics of participants will be described using appropriate summary statistics (parametric or non-parametric depending on the distribution of data) and compared between the intervention and control conditions. Comparisons will use paired-sample t-tests (continuous variables) or chi-square/Fishers Exact tests (categorical variables) or the equivalent non-parametric approaches.

### Interim Analysis (if applicable)

### N/A

### Handling of Missing Data

Missing data will be handled using standardized analytical approaches. Guidance will be sought from the project biostatistician (Dr. Carlson, U Pittsburgh). Where possible, missing data on the primary outcomes of interest (adult glycemic control, BMI, waist circumference, and blood pressure) will be abstracted from medical records.

# Trial Administration

## Ethical Considerations: Informed Consent/Assent and HIPAA Authorization

Enrollment into the proposed study will be voluntary. We will advertise the study in four Department of Education High Schools (see Research Strategy) and adolescents will reach out to study staff to express interest in participating. Adolescents and adults will then participate in a screening and consent process together; research staff will explain that we are interested in learning about whether adolescents can be equipped to support their family members in dealing with their diabetes. A general overview of what it entails to be in a research study and how randomization works will be reviewed with potential participants before a description of this specific study is provided. Specifically, we will explain to participants what this study would entail, explain that the adolescent would be the one to actually receive educational sessions, but that adults will continue with their usual care, and that we would be visiting their home three times over the course of the intervention to conduct research assessments. They will be informed that they will be enrolled in the study for a year, and will also be asked to provide feedback on the process during focus groups and/or semi-structured interviews. A description of all sources of materials to be collected at each assessment and throughout the intervention will be reviewed with the pair. Finally, they will be informed that each assessment will take about 40 minutes (each) for them to complete, and that they will be compensated $20 at each of the assessment visits (for $60 total) [this amount reflects the compensation recommended during preliminary discussions with the American Samoan IRB and was chosen to offset any opportunity costs associated with participation]. After this description of the study is completed, understanding will be checked (by the use of open ended questions and asking participants to describe in their own words what they are agreeing to), and any questions are answered, a trained member of the research team will obtain informed written consent from the participants. Consent forms will be Institutional Review Board (IRB)-approved. Adult participants will provide written consent, adolescents will provide written assent and biological parent consent (one parent) will be sought for the adolescent’s participation. The consent forms will be provided in both English and Samoan. Participants/LAR will be informed that participation is voluntary and that they may withdraw from the study at any time, without prejudice. A copy of the informed consent document will be given to the participants/LAR for their records.

## Institutional Review Board (IRB) Review

The study will be reviewed by both the Yale University IRB and the American Samoa Department of Health IRB. An IRB Authorization Agreement (IAA) will be established between Yale and The Miriam Hospital (Co-I Rochelle Rosen).

The protocol will be submitted to the primary IRBs (Yale and American Samoa) for review and approval. Approval of the protocol will be obtained before initiating any research activity. Any change to the protocol or study team will require an approved IRB amendment before implementation. The process for reporting unanticipated events/problems is described below. The IRBs will determine whether informed consent and HIPAA authorization are required.

A study closure report will be submitted to the IRBs after all research activities have been completed.

## Subject Confidentiality

Participant confidentiality and privacy is strictly held in trust by the participating investigators, their staff, and the sponsor(s) and their interventions. Therefore, the study protocol, documentation, data, and all other information generated will be held in strict confidence. No information concerning the study or the data will be released to any unauthorized third party without prior written approval of the sponsor.

Participants will be assigned identification numbers. Names of participants with linkage to the ID number will be kept in a password-protected file on our secure sever; only study staff will have knowledge of the password. Furthermore, consent forms and other forms with identifying information will be kept in locked file cabinets that are separate from storage of de-identified data files. Audio files from taped focus groups or semi-structured interviews will be immediately downloaded to our secure server, Yale Secure Box (and deleted from the audio recorder). Only the research assistants, Project Director, Health Educators and the PI will have access to patient identification. Given the nature of the intervention and focus group discussions, and the potential for breach of confidentiality by other participants, all participants will receive information about the importance of maintaining the confidence of other group members prior to participation. In the first sessions of the group meetings (intervention and control), adolescents will work together, guided by the facilitators, to establish their own rules for engagement and confidentiality. All participants will be asked to provide their signed agreement to those ‘rules’.

All key personnel have undergone/will undergo the mandatory Human Subjects Education Training required by their institutions prior to study commencement. All staff will complete mandatory FBI and child abuse clearances prior to being hired to work on the study. The Study PI and Co-I (Rosen) will work with the project director and the research assistants to train all study staff in collection of data. Staff will not be allowed to work with participants until they demonstrate proficiency in their role.

*Additional Protections for Children Involved as Subjects in Research*: As noted above, all potential participants will be formally consented/assented for the study only after receiving detailed, developmentally appropriate information about the study and having adequate time and opportunity to have their questions answered. The PI, Co-Is, and research team will proactively and promptly address any unanticipated harm and all investigators have had experience working with this age group.

The study monitor, representatives of the Institutional Review Board (IRB), or regulatory agencies may inspect all documents and records required to be maintained by the investigator, including but not limited to, medical records (office, clinic, or hospital) for the participants in this study. The clinical study site will permit access to such records.

Study participant research data, which is for purposes of statistical analysis and scientific reporting, will be transmitted to and stored at the Yale School of Public Health. This will not include the participant's contact or identifying information. Rather, individual participants and their research data will be identified by a unique study identification number. The study data entry and study management systems used will be secured and password protected. At the end of the study, all study databases will be de-identified and archived at YSPH.

## Deviations/Unanticipated Problems

A protocol deviation is any noncompliance with the study protocol. The noncompliance may be either on the part of the participant, the investigator, or the study site staff. As a result of deviations, corrective actions will be developed by the site and implemented promptly.

It will be the responsibility of the PI, Dr. Hawley, to identify and report deviations within five working days of identification of the protocol deviation. All deviations must be addressed in study source documents, reported to the study sponsor, and the reviewing Institutional Review Board (IRB) per their policies.

Unanticipated problems involving risks to participants or others include, in general, any incident, experience, or outcome that meets all of the following criteria:

- - - - - Unexpected in terms of nature, severity, or frequency given (a) the research procedures that are described in the protocol-related documents, such as the Institutional Review Board (IRB)-approved research protocol and informed consent document; and (b) the characteristics of the participant population being studied;
        - Related or possibly related to participation in the research (“possibly related” means there is a reasonable possibility that the incident, experience, or outcome may have been caused by the procedures involved in the research); and
        - Suggests that the research places participants or others at a greater risk of harm (including physical, psychological, economic, or social harm) than was previously known or recognized.

The investigator will report unanticipated problems (UPs) to the reviewing Institutional Review Board (IRB) and to the study sponsor. The UP report will include the following information:

- - - - - Protocol identifying information: protocol title and number, PI's name, and the IRB project number;
        - A detailed description of the event, incident, experience, or outcome;
        - An explanation of the basis for determining that the event, incident, experience, or outcome represents an UP;
        - A description of any changes to the protocol or other corrective actions that have been taken or are proposed in response to the UP.

To satisfy the requirement for prompt reporting, UPs will be reported using the following timeline:

- - - - - UPs that are serious adverse events (SAEs) will be reported to the IRB and study sponsor, if applicable within 48 hours of the investigator becoming aware of the event.
        - Any other UP will be reported to the IRB and study sponsor within five days of the investigator becoming aware of the problem.
        - All UPs will be reported to appropriate institutional officials (as required by an institution's written reporting procedures), the supporting agency head (or designee), and the Office for Human Research Protections (OHRP) within five days of the IRB's receipt of the report of the problem from the investigator.

## Data Quality Assurance

The Yale-based investigators (Drs. Hawley, Rivara, and Rabin) will provide standardized training to all research staff participating in data collection activities. Dr. Hawley will meet at least weekly with the American Samoa-based project director for the study. Standard Operating Procedures and an accompanying manual will be used for training and study implementation. Fidelity checklists will be created and fidelity reported for all group sessions delivered. Similar checklists for research assessments will be used and reported to the investigator team. The electronic nature of data collection, through REDCap, means that Yale-based researchers will be able to monitor data collection in real time.

## Study Records

Study records will include the following:

- Consent forms
- Responses to questionnaire measures and physical measurement generated during research assessments
- Medical records (adult participants only)
- Fidelity checklists from intervention sessions
- Transcripts of semi-structured interviews and focus groups

**Note: No biological specimens will be stored as part of this research.**

## Access to Source

Source data will be maintained per Medical Records policy in a password protected, secure, Health Insurance Portability and Accountability Act (HIPAA) compliant, web-based electronic database with a built-in audit trail.

Only Institutional Review Board (IRB) approved research team members who have current HIPAA and Collaborative Institutional Training Initiative (CITI) Good Clinical Practice (GCP) and human subjects protection training will be authorized to access records.

## Data or Specimen Storage/Security

## Consent forms will be stored as hard copies in a locked file cabinet, separate from any research data. Only the Project Director and study staff responsible for the project’s conduct will have access to consent forms. Upon completion of the study, consent forms will be transferred to Yale for long-term storage.

## Questionnaire data, medical record data, and physical measurements from research assessments (de-identified) and fidelity checklists will be captured by research assistants into REDCap, a Yale-approved, secure online platform. Access to the dataset will be restricted to those with Yale approval and IRB training. Database downloads and backups will be stored on Yale Secure Box.

## Focus groups and semi-structured interviews will be audio recorded. Immediately upon the completion of interviews, audio files will be uploaded to Yale Secure Box and the originals deleted from the recorders. When the audio files have been transcribed, translated, and back translated (within three months of original recording) the audio files will be permanently deleted.

## Retention of Records

Study data, including identifiable information, will be retained indefinitely. Identifying information will be stored to facilitate later follow up of the study participants if research findings suggest that doing so would be beneficial. Study data (de-identified) will be retained so that any queries resulting from publication of the study findings may be appropriately addressed.

## Study Monitoring

Study monitoring will be the responsibility of the PI, who will work with the co-investigators, project director and research staff to ensure study oversight.

## Study Modification

All proposed protocol modifications will be submitted to both the Yale and American Samoa Institutional Review Boards for approval prior to implementation. Both IRBs will need to give there permission before any change to the protocol is implemented.

## Study Completion

The study will be completed in September 2025. A completion report will be submitted to both the Yale and American Samoa IRB upon study closure.

## Funding Source

This study is funded by an R01 from the National Institutes of Health, National Institute for Diabetes, Digestive, and Kidney Diseases (NIDDK), PI: Dr. Hawley.

## Conflict of Interest Policy

The independence of this study from any actual or perceived influence, such as by the pharmaceutical industry, is critical. Therefore, any actual conflict of interest of persons who have a role in the design, conduct, analysis, publication, or any aspect of this trial will be disclosed and managed. Furthermore, persons who have a perceived conflict of interest will be required to have such conflicts managed in a way that is appropriate to their participation in the trial. The study leadership in conjunction with the appropriate conflict of interest review committee has established policies and procedures for all study group members to disclose all conflicts of interest and will establish a mechanism for the management of all reported dualities of interest.

At the time of submission, the investigators have no conflicts of interest to report. All investigators will follow the applicable conflict of interest policies*.*

## Publication Plan

Research study findings will be disseminated first to participants, then the American Samoa community, and finally in peer reviewed academic journals and at academic conferences. Publication and sharing of findings will proceed as the study progresses. Any publications/reports will be shared with the American Samoa Department of Health. The PI, Dr. Hawley, has primary responsibility for publishing the study results.

# Appendices

| **Appendix #** | **Title** | **Section** | **Topic** |
| --- | --- | --- | --- |

# List of Tables

**Table 1: Overview of the Proposed Intervention Curriculum**

**Table 2: Proposed Evaluation Activities**

# Bibliography

1. Centers for Disease Control. National diabetes statistics report, 2017: estimates of diabetes and its burden in the United States. Available at: <https://www.cdc.gov/diabetes/pdfs/data/statistics/national-diabetes-statistics-report.pdf> [Accessed October 23, 2019]
2. Rowley WR, Bezold C, Arikan Y, Byrne E, Krohe S. Diabetes 2030: Insights from yesterday, today, and future trends. Popul Health Manag 2017; 20(1): 6-12. **PMCID: PMC5278008**
3. American Diabetes Association. Economic costs of diabetes in the U.S. in 2017. Diabetes Care 2018; 41(5): 917-928. **PMCID: 5911784**
4. Agency for Healthcare Research and Quality. Diabetes disparities among racial and ethnic minorities fact sheet. <http://www.ahrq.gov/research/diabdisp.htm>. [Accessed September 19, 2019]
5. McElfish PA, Purvis RS, Esquivel MK, Sinclair KA, Townsend C, Hawley NL, Haggard-Duff LK, Kaholokula JK. Diabetes disparities and promising interventions to address diabetes in native Hawaiian and Pacific Islander populations. Curr Diab Rep 2019; 19 (5):19. PMID: 30887399
6. World Health Organization. American Samoa NCD Risk Factors: STEPS Report. 2007. <https://www.who.int/ncds/surveillance/steps/Printed_STEPS_Report_American_Samoa.pdf> [Accessed October 02, 2019]
7. Carlin M, Mendoza-Williams A, Ensign K. Half an ocean away: health in the US-affiliated Pacific Islands. J Public Health Manag Pract 2016; 22(5): 492-495. PMID: 27479309
8. Grieco E. The Native Hawaiian and other Pacific Islander population: Census 2000 brief. 2001. <http://www.census.gov/prod/2001pubs/c2kbr01-14.pdf> [Accessed September 19, 2019]
9. Bureau USC. 2010 Census Shows More than Half of Native Hawaiians and Other Pacific Islanders Report Multiple Races. 2010.
10. Hixson L, Hepler B, Kim M. The Native Hawaiian and Other Pacific Islander Population: 2010. 2012. <http://www.census.gov/prod/cen2010/briefs/c2010br-12.pdf> [Accessed September 19, 2019]
11. Glazier RH, Bajcar J, Kennie NR, Wilson K. A systematic review of interventions to improve diabetes care in socially disdvantaged populations. Diabetes Care 2006; 29(7): 1675-1688. PMID: 16801602
12. Noar SM, Benac CN, Harris MS. Does tailoring matter? Meta-analytic review of tailored print health behavior change interventions. Psychol Bull 2007; 133(4): 673-693. PMID: 17592961
13. Lagisetty PA, Priyadarshini S, Terrell S, Hamati M, Landgraf J, Chopra V, Heisler M. Culturally targeted strategies for diabetes prevention in minority populations: a systematic review and framework. Diabetes Educ 2017; 43(1): 54-77. **PMCID: PMC5408505**
14. Kaholokula JK, Wilson RE, Townsend CKM, Zhang GX, Chen J, Yoshimura SR, Dillard A, Yokota JW, Palakiko DM, Gamiao S, Hughes CK, Kekauoha BK, Mau Mk. Translating the Diabetes Prevention Program in Native Hawaiian and Pacific Islander communities: the PILI ‘Ohana Project. Transl Behav Med 2014; 4(2): 149-159. **PMCID: PMC4041922**
15. Simmons D, Fleming C, Voyle J, Fou F, Feo S, Gatland B. A pilot urban church-based program to reduce risk factors for diabetes among Western Samoans in New Zealand. Diabet Med 1998; 15(2): 136-142. PMID: 9507914.
16. Capstick S, Norris P, Sopoaga F, Tobata W. Relationships between health and culture in Polynesia – a review. Soc Sci Med 2009; 68(7): 1341-1348. PMID: 19195751
17. McMullin J. The call to life: revitalizing a healthy Hawaiian identity. Soc Sci Med 2005; 61(4): 809-820. PMID: 15950093
18. Finau SA. Health, environment and development: towards a Pacific paradigm. Pac Health Dialog 1996; 3(2): 266-278.
19. Stewart DW, DePue J, Rosen RK, Bereolos N, Goldstein M, Tuitele J, Nu’usolia O, McGarvey ST. Medication-taking beliefs and diabetes in American Samoa: a qualitative inquiry. Transl Behav Med 2013; 3(1): 30-38. **PMCID: PMC3607390**
20. Kaholokula JK, Ing CT, Look MA, Delafield R, Sinclair K. Culturally responsive approaches to health promotion for Native Hawaiians and Pacific Islanders. Ann Hum Biol 2018; 45 (3): 249-263. **PMCID: PMC6002761**
21. Kaholokula JK, Mau MK, Efird JT, Leake A, West M, Palakiko DM, Yoshimura SR, Kekauoha BP, Rose C, Gomes H. A family and community focused lifestyle program prevents weight regain in Pacific Islanders: a pilot randomized controlled trial. Health Educ Behav 2012; 39 (4): 386-395. **PMCID: PMC3154977**
22. McElfish PA, Long CR, Kohler PO, Yeary K, Bursac Z, Narcisse MR, Felix HC, Rowland B, Hudson JS, Goulden PA. Comparative effectiveness and maintenance of diabetes self-management education interventions for Marshallese patients with Type 2 Diabetes: a randomized controlled trial. Diabetes Care 2019; 42 (5): 849-858. **PMCID: PMC6489107**
23. Tolvanen M, Anttonen V, Mattila ML, Hausen H, Lahti S. Influence of children’s oral health promotion on parents’ behaviors, attitudes and knowledge. Acta Odontol Scand 2016; 74(5): 321-327. PMID: 26651375
24. Molnar A. Children as agents of change in combatting antibiotic resistance. J Health Serv Res Policy 2017; 22(4): 258-260. PMID: 28429983
25. Bresee S, Caruso BA, Sales J, Lupele J, Freeman MC. ‘A child is also a teacher’: exploring the potential for children as change agents in the context of a school-based WASH intervention in rural Eastern Zambia. Health Educ Res 2016; 31(4): 521-534. PMID: 27206442.
26. Gadhoke P, Christiansen K, Swartz J, Gittelson J. “Cause it’s family talking to you”: children acting as change agents for adult food and physical activity behaviors in American Indian households in the Upper Midwestern United States. Childhood 2015; 22(3): 346-361.
27. Williams O, DeSorbo A, Noble J, Gerin W. Child-mediated stroke communication: findings from Hip Hop Stroke. Stroke 2012; 43(1): 163-169. **PMCID: PMC3246577**
28. Laroche HH, Davis MM, Forman J, Palmisano G, Schacht Reisinger H, Tannas C, Spencer M, Heisler M. Children’s roles in parents’ diabetes self-management. Am J Prev Med 2009; 37(6): S251-S261. **PMCID: PMC2811065**
29. Ofahengaue V, Godinet, MT. Family and culture, and the Samoan youth. J Fam Soc Work 2008; 11: 229-253.
30. Smyth C, Cass B, Hill T. Children and young people as active change agents in care-giving: agency and constraint. Children Youth Services Review 2011; 33: 509-514.
31. Groop L, Forsblom C, Lehtovirta M, et al. Metabolic consequences of a family history of NIDDM (the Botnia study): evidence for sex-specific parental effects. Diabetes 1996; 45(11): 1585–1593. PMID: 8866565
32. Meigs JB, Cupples LA, Wilson PW. Parental transmission of type 2 diabetes: the Framingham Offspring Study. Diabetes 2000; 49(12): 2201–2207. PMID: 11118026
33. Pettitt DJ, Talton J, Dabelea D, Divers J, Imperatore G, Lawrence JM, Liese AD, Linder B, Mayer-Davis EJ, Pihoker C, Saydah SH, Standiford DA, Hamman RF, SEARCH for Diabetes in Youth Study Group. Prevalence of diabetes in U.S. youth in 2009; the SEARCH for diabetes in youth study. Diabetes Care 2014; 37(2): 402-408. **PMCID: PMC3898760**
34. Boyle JP, Honeycutt AA, Venkat Narayan KM, Hoerger TJ, Geiss LS, Chen H, Thompson TJ. Projection of diabetes burden through 2050: impact of changing demography and disease prevalence in the U.S. Diabetes Care 2001; 24(11): 1936-1940. PMID: 11679460
35. TODAY Study Group, Zeitler P, Hirst K, Pyle L, Linder B, Copeland K, Arslanian S, Cuttler L, Nathan DM, Tollefsen S, Wilfley D, Kaufman F. A clinical trial to maintain glycemic control in youth with Type 2 Diabetes. N Engl J Med 2012; 366(24): 2247-2256. **PMCID: PMC3478667**
36. TODAY Study Group. Retinopathy in youth with Type 2 Diabetes participating in the TODAY clinical trial. Diabetes Care 2013; 36(6): 1772-1774. **PMCID: PMC3661825**
37. US Census Bureau. American Samoa Demographic Profile Data. 2010. www.factfinder.census.gov/faces/tableservices/jsf/pages/productview.xhtml?pid=DEC_10_DPAS_A

SDP1&prodType=table [Accessed October 03, 2019]

1. Hawley NL, Weir LM, Cash HL, Viali S, Tuitele J, McGarvey ST. Modernization and cardiometabolic risk in Samoan adolescents. Am J Hum Biol 2012; 24(4): 551-557. **PMCID: PMC3705767**
2. Department of Health, Department of Education, American Samoa. Prevalence of obesity in American Samoan Schoolchildren. <https://www.ctahr.hawaii.edu/adap/ASCC_LandGrant/Dr_Brooks/TechRepNo59.pdf> [Accessed October 31, 2019]
3. DePue JD, Rosen RK, Batts-Turner M, Bereolos N, House M, Forster Held R, Nu’usolia O, Tuitele J, Goldstein MG, McGarvey ST. Cultural translation of interventions: Diabetes Care in American Samoa. Am J Public Health 2010; 100(11): 2085-2093. **PMCID: PMC2951958**
4. DePue JD, Goldstein MG, Dunsiger S, Nu’usolia O, Seiden AD, Tuitele J, Blume J, McGarvey ST, Rosen RK. Nurse-community health worker intervention improves diabetes care in American Samoa: results of a randomized controlled trial. Diabetes Care 2013; 36(7): 1947-53. **PMCID: PMC3687286**
5. Hamid S, Dunsiger S, Seiden A, Nu’usolia O, Tuitele J, DePue JD, McGarvey ST. Impact of a diabetes control and management intervention on health care utilization in American Samoa. Chronic Ill 2014; 10(2): 122-134. **PMCID: PMC4218844**
6. Huang SJ, Galarraga O, Smith KA, Fuimaono S, McGarvey ST. Cost-effectiveness analysis of a cluster-randomized, culturally tailored, community health worker home-visiting diabetes intervention versus standard care in American Samoa. Hum Res Health 2019; 17(1): 17. **PMCID: PMC6402127**
7. Gary TL, Bone LR, Hill MN, Levine DM, McGuire M, Saudek C, Brancati FL. Randomized controlled trial of the effects of nurse case manager and community health worker interventions on risk factors for diabetes-related complications in urban African Americans. Prev Med 2003; 37(1): 23-32. PMID: 12799126
8. Gary TL, Batts-Turner M, Yeh H, Hill-Briggs F, Bone LR, Wang NY, Levine DM, Powe NR, Saudek CD, Hill MN, Brancati FL. The effects of a nurse case manager and a community health worker team on diabetic control, emergency department visits, and hospitalizations among urban African Americans with type 2 diabetes mellitus: A randomized controlled trial. Arch Intern Med 2009; 169:(19): 1788–1794. **PMCID: PMC5675128**
9. Rao M, DePue JD, Dunsiger S, Elsayed M, Nu’usolia O, McGarvey ST. Long-term impact of a community health worker intervention on diabetes control in American Samoa. Prev Chronic Dis 2-15; 12:E180. **PMCID: PMC4617456**
10. Ashman A, Gillies R. Cooperative learning: The social and intellectual outcomes of learning in groups. Abingdon: Routledge; 2003
11. Bransford, J.D., Brown, A.L., and Cocking, R.R. (Eds.) (1999). How people learn: Brain, mind, experience, and school. Washington, D.C.: National Academy Press.
12. Cunningham SD, Lewis JB, Thomas JL, Grilo SA, Ickovics JR. Expect With Me: development and evaluation design for an innovative model of group prenatal care to improve perinatal outcomes. BMC Pregnancy Childbirth 2017; 17(1): 147. **PMCID: PMC5437650**
13. Brame CJ, Biel R. Setting up and facilitating group work:
    Using cooperative learning groups effectively. 2015. <http://cft.vanderbilt.edu/guides-sub-pages/setting-up-and-facilitating-group-work-using-cooperative-learning-groups-effectively/> [Accessed October 09, 2019]
14. Kanaya AM, Adler N, Moffet HH, Liu J, Schillinger D, Adams A, Ahmed AT, Karter AJ. Heterogeneity of diabetes outcomes among asians and pacific islanders in the US: the diabetes study of northern california (DISTANCE). *Diabetes Care.* 2011; 34(4):930-937. **PMCID: PMC3064053**
15. Mau MK, West MR, Shara NM, Efird JT, Alimineti K, Saito E, Sugihara J, Ng R. Epidemiologic and clinical factors associated with chronic kidney disease among Asian Americans and Native Hawaiians. Ethn Health 2007; 12(2):111-127. PMID: 17364897
16. McElfish P, Moore R, Woodring D, Purvis RS, Maskarinec GG, Bing WI, Hudson J, Kohler PO, Goulden PA. Social ecology and diabetes self-management among Pacific Islanders in Arkansas. J Fam Med Dis Prev 2016; 2(1):026. **PMCID: PMC5518699**
17. Hallgren E, McElfish P, Rubon-Chutaro J. Barriers and opportunities: a community-based participatory research study of health beliefs related to diabetes in a US Marshallese community. Diabetes Educ. 2015; 41(1):86-94. **PMCID: PMC4406271**
18. McElfish P, Hallgren E, Yamada S. Effect of US health policies on health care access for Marshallese migrants. Am J Public Health 2015; 105(4):637-643. **PMCID: PMC4358182**
19. Juarez D, Davis J, Brady S, Chung R. Prevalence of heart disease and its risk factors related to age in Asians, Pacific Islanders, and Whites in Hawai'i. J Health Care Poor Underserved 2012; 23(3):1000-1010. **PMCID: PMC5677527**
20. Juarez DT, Samoa RA, Chung RS, Seto TB. Disparities in health, obesity and access to care among an insured population of Asian and Pacific Islander Americans in Hawai'i. *Hawaii Med J.* 2010; 69(2):42-46. **PMCID: PMC3104634**
